# Intrinsic basis of thermostability of prolyl oligopeptidase from *Pyrococcus furiosus*

**DOI:** 10.1038/s41598-021-90723-4

**Published:** 2021-06-02

**Authors:** Sahini Banerjee, Parth Sarthi Sen Gupta, Rifat Nawaz Ul Islam, Amal Kumar Bandyopadhyay

**Affiliations:** 1 Department of Biological Sciences, Indian Statistical Institute, Kolkata, West Bengal India; 2 Department of Chemical Sciences, Indian Institute of Science Education and Research, Berhampur , Orissa India; 3 Department of Zoology, The University of Burdwan, Burdwan, West Bengal India; 4 Department of Biotechnology, The University of Burdwan, Burdwan, West Bengal India

**Keywords:** Biochemistry, Biophysics, Biotechnology, Computational biology and bioinformatics, Genetics, Structural biology

## Abstract

Salt-bridges play a key role in the thermostability of proteins adapted in stress environments whose intrinsic basis remains to be understood. We find that the higher hydrophilicity of PfP than that of HuP is due to the charged but not the polar residues. The primary role of these residues is to enhance the salt-bridges and their ME. Unlike HuP, PfP has made many changes in its intrinsic property to strengthen the salt-bridge. First, the desolvation energy is reduced by directing the salt-bridge towards the surface. Second, it has made bridge-energy more favorable by recruiting energetically advantageous partners with high helix-propensity among the six possible salt-bridge pairs. Third, ME-residues that perform intricate interactions have increased their energy contribution by making major changes in their binary properties. The use of salt-bridge partners as ME-residues, and ME-residues' overlapping usage, predominant in helices, and energetically favorable substitution are some of the favorable features of PfP compared to HuP. These changes in PfP reduce the unfavorable, increase the favorable ME-energy. Thus, the per salt-bridge stability of PfP is greater than that of HuP. Further, unfavorable target ME-residues can be identified whose mutation can increase the stability of salt-bridge. The study applies to other similar systems.

## Introduction

The physical and chemical environments of the earth show a wide variation in which microorganism lives in almost all places. Depending on the temperature, the microbes living in these environments can be divided into three classes namely thermophiles (45–65 °C), mesophiles (25–45 °C), and psychrophiles (0–25 °C)^1^. Thermophiles operate their biochemical machinery at boiling temperatures that are detrimental to mesophilic microbes^2–4^. In the environment in which these microbes live when replicated in the laboratory, they and their proteins function optimally^5,6^. Although the topology of the optimized state of extremophilic proteins is similar to that of mesophiles due to the influence of the external environment and weak interactions, their homologous positions in the primary sequence are very different^7,8^. In other words, the biological specificity of such marginally stable proteins from mesophiles is not different from that operating in a stressful environment^8,9^. Due to the tireless efforts of the past years, our knowledge of protein thermostability has become much wider today. Proteins are usually made up of 20 acids, some of which are hydrophilic and some hydrophobic. Due to the compositional variation of amino acids, a wide variation of overall hydrophilicity and hydrophobicity can be observed in the functionally identical proteins in different environments. Similarly, biomass, which is composed of biomaterials other than proteins, shows wide-scale variations of these properties. Knowledge of this variation of proteins on the one hand makes it easier to understand its adaptability, on the other hand, it enhances its analytical and industrial applications^10^. There are several reasons for protein thermostability, such as favorable contributions of certain residues in the loops^11,12^, reduction of loops at the protein surface^13^, helix and hydrogen-bond stability^12,14^, especially alternative packing and compaction of amino acid residues in the surface regions^4,15–17^ increased polar surface^11,18^ and ion-pair^3,12,19–25^. By novel and expert substitution of the specific amino acid residue in the primary sequence of the thermophilic protein, it is possible to increase the protein thermostability on the one hand, and on the other hand, it is possible to understand the cause of the thermostability^22,26–30^.

Prolyl oligopeptidase is a Ser type protease located in the cytoplasm that is twice the size of conventional proteases like trypsin, pepsin, etc.^31,32^. This enzyme acts on peptide hormones and neuropeptides to make them functional. Its active site is protected by the beta-propeller domain and, therefore, can only act on small peptides (≤ 30 amino acids)^31–33^. The peptide bond on the C-terminal side of the Proline, attached to the trans-peptide bond, is the sessile bond for the enzyme. Naturally, a variety of clinical situations is caused due to the lack of functional enzyme or its abnormal action^31–35^. For these reasons, the enzyme has immense importance as a therapeutic target. Today, it has been a challenging job to make mesophilic homologue functional in various physical and chemical environments through protein engineering^2,32^. Thus, it is very challenging to understand the effectiveness of PfP at high temperatures and to increase the robustness of its orthologous enzymes via protein engineering.

In comparison to HuP, reduction of the loops and increase in the frequency of the salt-bridge have been the cause of thermostability of the PfP^19,24,33^. The energy contribution of salt-bridge and microenvironment in this context is still unknown. These energies can be extracted by the solution of the PBE. Salt-bridge is usually of two types, namely IP and NU. net (*ΔΔG*_*net*_) and component (desolvation: *ΔΔG*_*dslv*_, bridge: *ΔΔG*_*brd*_ and background: *ΔΔG*_*bac*_) energy terms of salt-bridge are extracted by applying IPM in the case of the former^36–39^ and NUM in the case of the latter^40^. Desolvation of salt-bridge residues is a thermodynamically uphill process and thus, *ΔΔG*_*dslv*_ is always positive. On the other hand, the interaction energy (*ΔΔG*_*brd*_) of positive and negative charges is always negative. *ΔΔG*_*bac*_, on the other hand, which interacts with salt-bridge's partners and whose candidates are originating from the sequence, maybe favorable or unfavorable^40,41^. The amount of *ΔΔG*_*net*_ was found to be highly favorable in the case of hyperthermophilic glutamate dehydrogenase than that of its mesophilic counterpart^36,42^. Nevertheless, the intrinsic basis of protein thermostability^2^ remains unknown today. Here, the knowledge of the microenvironment (ME) of protein seems to be central^40^. Notably, while *ΔΔG*_*dslv*_ and *ΔΔG*_*brd*_ provide partners of salt-bridge specific details, *ΔΔG*_*bac*_ is related to other residues of protein^36–40,42^. A ME of a salt-bridge is the residues that are interacting with the partners of salt-bridge beyond four Å distance (in the case of charged residues) under a given cut-off of the energy. Thus, the ME of a protein refers to the collection of all these residues of salt-bridges of the protein. Analyses of ME-residues in terms of the interaction-energy, residue composition, and class, type of secondary structure, and core or surface location may reveal insights pertaining to the protein thermostability.

Here, we have tried to understand the above-mentioned concerns through a detailed analysis of the PfP sequence and structure relative to HuP. The relationship of salt-bridge and microenvironments energetics and other properties with the property in the underlying sequence is presented in this work. Application of NUM method for network salt-bridge and microenvironment can also be found in this study. Further, the application and effectiveness of residue-specific microenvironment energy and its binary details in site-directed mutations are also shown in our study. Taken together, we think our work indeed will play a significant role in understanding targeted protein engineering and protein thermostability.

## Results

### The properties of the homologous positions in the sequence of HuP and PfP

**Figure 1:**
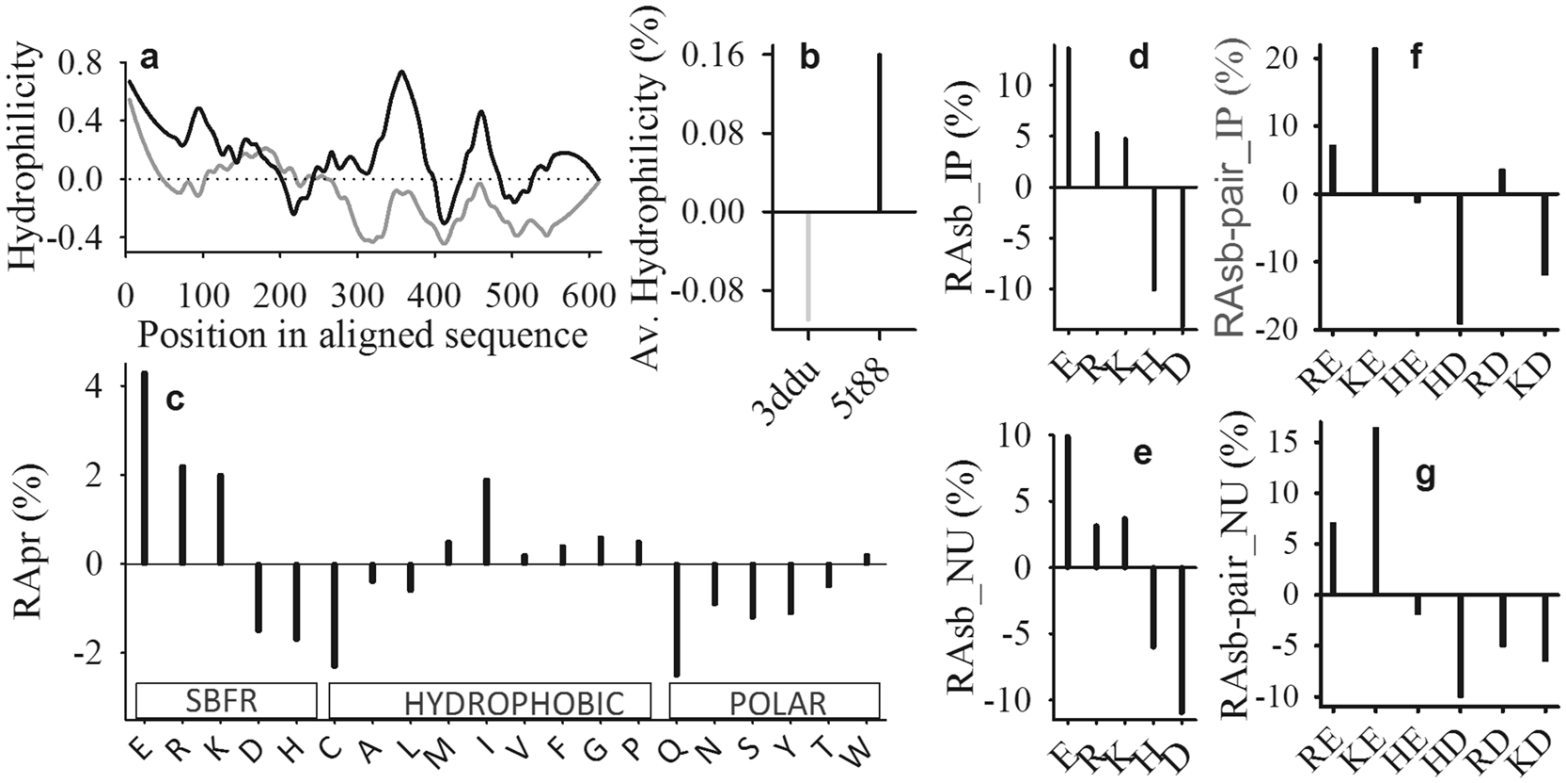
PfP has higher hydrophilicity due to the higher abundance of salt-bridge forming residues (SBFR). Hydrophilicity profiles (**a**) and grand average hydrophilicity (**b**) of PfP (black) and HuP (grey). Normalized relative abundance (RApr in %) of salt-bridge forming residues (charged), hydrophobic, and polar classes of amino acids (**c**) of the sequence of PfP in reference to HuP. Normalized relative abundance (in %) of salt-bridge forming residues for isolated pair (**d**) and network unit (**e**) types of salt-bridges of PfP in reference to HuP. The normalized relative abundance of pair-types of salt-bridge for isolated-pair (f) and network unit (**g**) of PfP in reference to HuP.

A comparative analysis of HuP (Human oligopeptidase) and PfP's (*Pyrococcus furiosus* oligopeptidase) sequences and their high-resolution crystal structures, 3ddu (resolution, 1.56 Å) and 5t88 (resolution, 1.90 Å), respectively, is the focus of the study. The Hopp and Woods hydrophilicity^43^ profile (Fig. 1a) on T-coffee aligned sequence block^44^ comparing the hydrophilicity of the aligned sequence (Supplementary Figure S1). It shows that the hydrophilicity of almost every position in the sequence of PfP is higher than that of HuP (Fig. 1a). Similarly, the value of grand average hydrophilicity is positive in the case of PfP, but in the case of HuP, it is negative (Fig. 1b, Supplementary Table S2). Here, we expected that the normalized relative frequency of the polar residue (i.e. N, Q, S, T, and Y) in the sequence of PfP would be higher than that of HuP. In contrast, we see the higher and lower normalized relative frequency of selected charged (E, R, and K) and polar residues in the case of PfP, respectively (Fig. 1c and Supplementary Table S3). The pattern of relative abundance in these charged residues (E, R, K, D, H) participating in IP (Fig. 1d) and NU (Fig. 1e) has been similar (Supplementary Table S4) to that of the sequence (Fig. 1c). Further, subject to HuP, the relative abundance of the charged residues of the sequence of PfP (Fig. 1c) is strongly correlated with that of the IP (Fig. 1d) and NU (Fig. 1e). Moreover, out of RE, RD, KE, KD, HE, and HD possible pairs of salt-bridge, only RE, KE, and RD show positive relative abundance for IP (Fig. 1f, Supplementary Table S5) and RE and KE for NU (Fig. 1g, Supplementary Table S5) types of salt-bridges of PfP.

Table 1 describes the participation of PfP and HuP charged residues in IP and NU salt-bridge. In the former case, the frequency of R, K, and E in IP and NU, is higher than in the latter. In PfP, the 56 and 53 charged residues form 28 IPs and 16 NUs (i.e. 37 pairs) types salt-bridge, respectively, which is much less in the case of HuP (Table 1). Although the number of salt-bridges in PfP is higher, the frequency of buried salt-bridges is much less (34%) than that of HuP (45%). 56% (109 of 196) and 44% (83 of 189) of the total charge residue in the proteins of PfP and HuP are present in the salt-bridge, respectively (Table 1).

**Table 1:** Salt-bridge forming residues constitute only about half of the total charged residues.

Res type	5t88 (616 residues)				3ddu (707 residues)			
	IP Res	NU Res	SB Res	Charged Res	IP Res	NU Res	SB Res	Charged Res
R	11	12	23	35	6	8	14	25
K	16	11	27	53	10	7	17	47
H	1	2	3	9	5	4	9	23
E	17	17	34	62	7	9	16	41
D	11	11	22	37	14	13	27	53
Total	56 (28.5%)	53 (27.0%)	109	196	42 (22.2%)	41 (21.6%)	83	189
Buried Res	–	–	37 (34%)	–	–	–	37 (45%)	-
SB pair	28	37 (16 NU)	–	–	21	30 (12 NU)	–	-

Absolute frequency of isolated pair residue (IP Res), network unit residue (NU Res), and charged residue of 5t88 (left) and 3ddu (right). Total salt-bridge residues (SB Res) is the sum of IP Res and NU Res. The buried residue (Buried res) is the buried residues.

This means that the rest of the charged residues are present in different locations of the protein as the isolated charge. How do these isolated charges (along with the polar residue) relate to the structure and stability of the protein? Is there any uniqueness or difference between PfP and HuP in this context?

### The electrostatic strength of each salt-bridge of HuP and PfP

**Figure 2:**
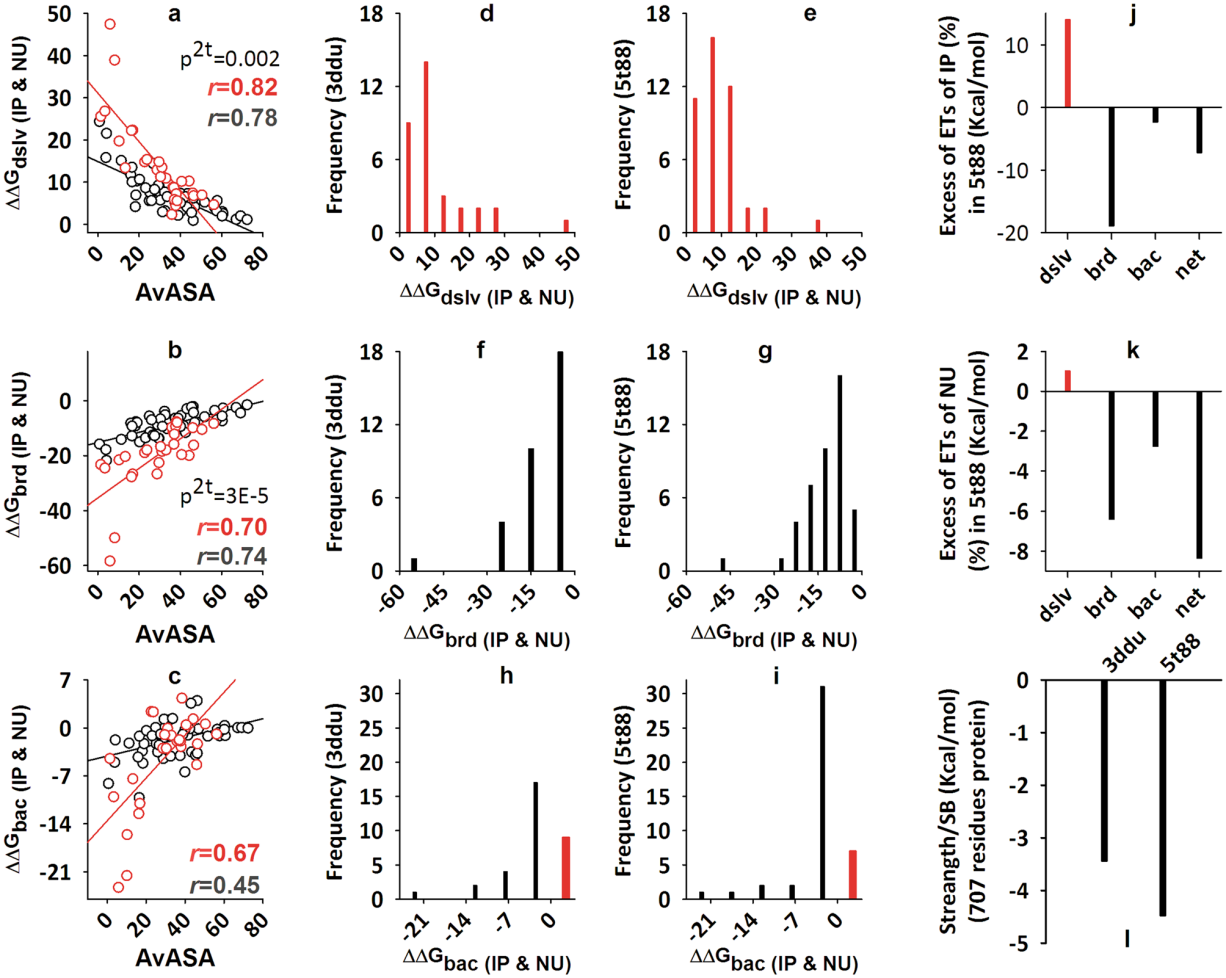
Component and net energy terms for isolated and network types of salt-bridge are more favorable in 5t88 than 3ddu. Correlation plot of *ΔΔG*_*dslv*_ (**a**), *ΔΔG*_*brd*_ (**b**) and *ΔΔG*_*bac*_ (**c**) with ASAav for IP (black) and NU (red) salt-bridges (p^2t^ is 2tailed probability for a test of significance). Distribution of 5t88 and 3ddu's desolvation-energy (**d**,**e**), bridge-energy (**f**,**g**), and background-energy (**h**,**i**). Here, candidates of IP and NU are plotted together. Excess amount of 5t88's IP (**j**) and NU's (**k**) energy terms (ETs) relative to 3ddu. Normalized electrostatic strength of 5t88 and 3ddu subject to each salt-bridge (**l**).

The APBS solver method^45^ is very popular for the extraction of the energy of the salt-bridge^39^. Similar to previous studies on salt-bridge^36–38^, we used the APBS method, and parameters (Supplementary Table S6) to extract the energy for IP (Supplementary Tables S7, S8 for 3ddu and Supplementary Table S9 for 5t88) and NU (Supplementary Tables S7, S10 for 3ddu and Supplementary Table S11 for 5t88) salt-bridge by IPM^36–39^ and NUM^40^, respectively. The salt-bridge of the protein is located at different locations (core or surface) of the protein. Salt-bridge partner's ASA shows that in the case of 5t88 and 3ddu, 67% and 58% of the partners, respectively, exist on the surface. Since the dielectric constant is location-dependent, it was necessary to investigate how the component energy terms are affected by this parameter. The apparent linear correlation of the energy terms is following the order as *ΔΔG*_*dslv*_ > *ΔΔG*_*brd*_ > *ΔΔG*_*bac*_. *ΔΔG*_*dslv*_ is inversely and *ΔΔG*_*brd*_ is directly, linearly and significantly related to ASAav (Fig. 2a,b). Such a correlation appears uncertain in the case of *ΔΔG*_*bac*_ (Fig. 2c). In all these cases, the curve for the NU (red) is steeper than that of the IP (black) salt-bridge (Fig. 2a,b). Notably, a network unit is composed of three or more salt-bridge partners.

In both 5t88 and 3ddu, the distribution of energy terms implies that desolvation energy is always costly (Fig. 2d,e; Supplementary Tables 8, 9, 10, 11) and bridge energy, in turn, (Fig. 2f,g; Supplementary Tables 8, 9, 10, 11) is always favorable. Here, the background term is a combination of more favorable and less unfavorable candidates (Fig. 2h,i; Supplementary Tables 8, 9, 10, 11). Comparing these energy terms between 5t88 (Supplementary Tables 9, 11) and 3ddu (Supplementary Tables 8, 10), we see some interesting observations. First, although in the case of 5t88 (Fig. 2e), the frequency of candidates with low desolvation cost (≤ 20 kcal/mol) is higher; in the case of 3ddu (Fig. 2d), such an observation with desolvation energy > 20 kcal/mol is also true. Second, interestingly, in the case of 5t88, the frequency of the candidates in the favorable bridge term is much higher than that of 3ddu (Fig. 2f,g). Overall, in the case of 5t88, the desolvation energy is 62.3 kcal/mol more unfavorable than that of 3ddu. At the same time, in 5t88, the bridge energy is − 119.6 kcal/mol more favorable than in 3ddu (Supplementary Table S7). This means that from these two energy terms, in 5t88, excess gain in favorable energy is − 57.2 kcal/mol. Desolvation and bridge energies tend to neutralize each other's effect, so, this much energy gain (− 57.2 kcal/mol of protein) from these two terms is very important for 5t88 in relation to its thermostability. Along with this, 5t88 further gained − 19.4 kcal/mol of additional energy from the *ΔΔG*_*bac*_, subject to 3ddu (Supplementary Table S7).

Figure 2j,k have been presented to understand this gain of energy terms on the normalized scale (per 100-residue protein) for the IP and NU salt-bridges in 5t88 relative to 3ddu. Although the relative gains of 5t88's IP and NU's net energy are almost equal (~ − 8.0 kcal/mol per 100 residues protein), they have not been achieved in the same way. In the case of IP (Fig. 2j), the relative excess of the unfavorable desolvation energy (red bar) has been mitigated largely by the favorable bridge-energy. In the case of NU (Fig. 2k), on the one hand, the desolvation energy is negligible, on the other hand, the effect of the favorable background energy is greater. It turns out that the electrostatic strength of each of 5t88's salt-bridges is greater by ~ − 1.04 kcal/mol than that of 3ddu's (Fig. 2l). Therefore, we see that in the former not only the number of salt-bridges is greater; in this case, the electrostatic strength per salt-bridge is also greater. Here, increasing the strength of each salt-bridge seems to be an underlying strategy. In this context, it should be mentioned that the count of surface-directed salt-bridges in 5t88 (66%) is higher than that of 3ddu (Table 1), which reduces the desolvation energy^36^. It is also mentioned here that although each pair of salt-bridge is equally likely to contribute to protein stability^46^, preferred pairs that contribute more to stability^47^ and promote helix^48^ (i.e. KE and RE, and to some extent RD), are dominated in the salt-bridge population of 5t88 (Fig. 1f,g). Overall, as the number of salt-bridges increases, the electrostatic strength of the salt-bridge of PfP exceeds that of the HuP, indicating an intrinsic strategy.

### HuP, and PfP's salt-bridge microenvironment energetics and binary properties

**Figure 3:**
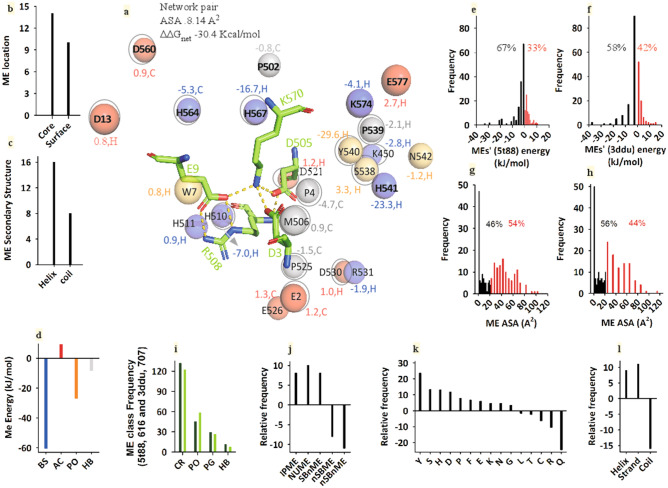
5t88 than 3ddu's microenvironment of salt-bridge, its residue-class, distribution, and binary details are more promising. Typical ME (**a**, residues: acidic, red; basic, blue; polar, yellow; hydrophobic, grey; core residues are with a sphere with a ring; interaction-energy is shown in the same color of residue with secondary structure i.e. H, helix; C, coil) of a salt-bridge and its location (**b**), secondary structure (**c**) and interaction energy (**d**). The ME is present with the NU unit (ASAav 8.14 Å^2^ and net stability − 30.4 kcal/mol). Distribution of 5t88 (**e**) and 3ddu's (**f**) stable (black) and unstable (red) ME-population (IP & NU population together). Similarly, the distribution of 5t88 (**g**) and 3ddu's (**h**) buried (black) and exposed (red) ME-population (IP & NU population together). Normalized frequency of residue classes (charged, CR; polar PO; Pro and Glu PG and hydrophobic, HB) of 5t88 (black bar) and 3ddu's (green bar) ME-population (**i**). Relative frequency of charged ME-residues in 5t88 relative to 3ddu (**j**). IPME ME-residues that act as the microenvironment of the IP only, NUME ME-residues that act as the microenvironment of the NU only, SBnME SB-residues that do not act as the microenvironment, nSBME ME-residues that act as the microenvironment but not participate in salt-bridge and charged-residues that neither participate in the microenvironment nor the salt-bridge formation. Relative frequency of 5t88's ME-residues relative to 3ddu (**k**). Relative preference to the secondary structure of 5t88's ME-residues relative to 3ddu (**l**).

The length of HuP's 3ddu is 94 residues more than PfP's 5t88 (Supplementary Figure S1), but it's 22.2% (as IP) and 21.7% (as NU) of the charged residues participate in the salt-bridge (Table 1). In the case of 5t88, on the other hand, 28.5% (as IP) and 27.0% (as NU) of the charged residues participate in the salt-bridge (Table 1). The rest of the non-salt-bridge charged-residue (nSBME) remains as isolated charge in the protein. In addition to these, other types of residue (PO, PG, HB), IPME, NUME also participate as ME. A highly stable (− 30.4 kcal/mol) typical core (ASAav 8.14 Å^2^) and network salt-bridge's ME of 5t88 is presented in Fig. 3a. The ME environment is composed of isolated charged (red, acidic and blue, basic), polar (yellow), and hydrophobic (grey) residues (Fig. 3a). Although the number of hydrophilic residues (charged and polar) in ME is large, most of them are located in the core along with the core salt-bridge (Fig. 3b). Interestingly, two-third of the ME is located in the helix (Fig. 3c). Acidic residue's interaction is seen to be somewhat unfavorable but for all other types of ME-residue (BS, PO, HB), interaction energy is favorable (Fig. 3d).

Overall, the favorable population of ME of 5t88 (616 residues) is much higher than that of 3ddu (707 residues) (Fig. 3e,f). However, the core ME-population of 5t88 is less than that of 3ddu (Fig. 3g,h). Charged residues (CR), which are followed by polar (PO), PG, and hydrophobic (HB) (Fig. 3i) constitute the majority of this total ME-population. Charged residues can be broadly divided into five different categories such as IPME, NUME, SBnME, nSBME, and nSBnME. The population of the first three categories is higher in 5t88 than that of 3ddu (Fig. 3j). Although the polar-class is less in the sequence of 5t88 relative to 3ddu (Fig. 1c), from a ME-population point of view, this is not true for all these residues (Fig. 3k). 5t88's ME-population is higher in helix and strand relative to that of 3ddu (Fig. 3l). These features of ME-population of 5t88 and 3ddu are shown in detail in Tables 2 and 3 in the quantitative form (see below).

**Table 2:** Classes, categories, binary details, and interaction energies of microenvironment residues of 5t88.

ME-class of 5t88 (616 Res)	ME for SB-type	ME candidate type and count			Total count	Core count	Surface count	Energy kJ/mol	H	S	C	TU
		nSBME	IPME	NUME								
Charged (CR)	IP	20	9	8	37	6	31	− 34.50	10	13	14	47
	NU	16	15	13	44	13	31	− 80.11	19	7	18	59
	IP & NU	22	13	16	51	20	31	− 72.38	21	16	14	134
Polar (PO)	IP	22	–	–	22	17	5	− 104.08	3	11	8	23
	NU	21	–	–	21	12	9	− 101.65	10	5	6	21
	IP & NU	2	–	–	2	2	0	− 25.44	1	1	0	4
PRO & GLY (PG)	IP	15	–	–	15	11	4	− 4.89	1	3	11	16
	NU	11	–	–	11	8	3	− 19.46	4	0	7	14
	IP & NU	3	–	–	3	3	0	1.13	1	0	2	7
Hydrophobic (HB)	IP	6	–	–	6	4	2	− 12.41	1	5	0	6
	NU	5	–	–	5	4	1	1.96	2	0	3	5
	IP & NU	0	–	–	0	0	0	0.00	0	0	0	0
Grand total		143	37	37	217	100	117	− 451.84	73	61	83	336

The energy cut-off was ± 0.75 kJ/mol. ME-energy of IP (isolated pair) and NU (network pair) salt-bridge was extracted using IPM and NUM method, respectively. *H* ME count in helix, *S* ME count in strand, *C* ME count in coil, *TU* times used.

**Table 3:** Classes, categories, binary details, and interaction energies of microenvironment residues of 3ddu.

ME-class of 3ddu (707 Res)	ME for SB-type	ME candidate type and count			Total count	Core count	Surface count	Energy kJ/mol	H	S	C	TU
		nSBME	IPME	NUME								
Charged (CR)	IP	18	7	10	35	15	20	18.86	9	12	14	37
	NU	25	13	10	48	14	34	− 12.67	13	16	19	67
	IP & NU	23	9	7	39	19	20	− 136.90	12	6	21	144
Polar (PO)	IP	27	–	–	27	21	6	− 115.97	10	7	10	28
	NU	26	–	–	26	19	7	− 54.70	11	5	10	28
	IP & NU	5	–	–	5	5	0	− 23.41	1	1	3	12
PRO & GLY (PG)	IP	7	–	–	7	6	1	− 3.98	2	0	5	10
	NU	17	–	–	17	16	1	− 24.67	3	2	12	20
	IP & NU	2	–	–	2	2	0	− 3.60	1	0	1	5
Hydrophobic (HB)	IP	4	–	–	4	3	1	− 8.12	1	1	2	4
	NU	3	–	–	3	3	0	1.88	1	0	2	3
	IP & NU	0	–	–	0	0	0	0.00	0	0	0	0
Grand total		157	29	27	213	123	90	− 363.28	64	50	99	358

The energy cut-off was ± 0.75 kJ/mol. ME-energy of IP (isolated pair) and NU (network pair) salt-bridge was extracted using IPM and NUM method respectively. *H* ME count in helix, *S* ME count in strand, *C* ME count in coil; TU times used.

ME interaction energy was extracted using the APBS method^45^ either under default or mild parameters conditions (Supplementary Table 6) to avoid non-linearity related to the PBE. In the case of 5t88 and 3ddu, the energetics and binary details of these ME-residues have been described in Table 2 for 5t88 and Table 3 for 3ddu. Further, residue-specific details of ME-energy and binary properties are also shown in the supplementary tables (Supplementary Table S12 for 5t88 and Supplementary table S13 for 3ddu). Here, it is noteworthy that the interaction between the ME-candidate and the salt-bridge partner (acidic or basic) is very intricate, overlapping, interlinked, and complex that takes place across the detailed 3D-space of the protein. Many points are worth noting here. First, there are three categories of ME-residues for each residue-class (CR, PO, PG, and HB) such as ME for IP salt-bridge only, ME for NU salt-bridge only, and ME for both IP and NU types of salt-bridge. An IP or NU salt-bridge partner (acidic or basic) may act as a ME-candidate for other IP or NU types of salt-bridge. Second, although the residues in classes other than the charged-class are always of the non-salt-bridge type, they can participate as ME-candidates in various IP and NU salt-bridges in an overlapping and exhaustive manner (Supplementary Table S12 for 5t88 and Supplementary table S13 for 3ddu). Third, although the number of residues in 5t88 is lower (616 residues) than that of 3ddu (707 residues), the total count of residues in ME is almost the same i.e. 213 and 217 respectively. In the former, the number is higher in the ME-residue of the partner of salt-bridge (IPME and NUME), but in the latter case, the number is higher in the ME-residue of nSBME nature. Fourth, in 5t88, a greater number of ME-residues are located at the surface (Table 2; Fig. 3g), in contrast, in 3ddu, the greater the number of ME-residues are present at the core (Table 3; Fig. 3h). While most of the 5t88's ME-residues are in helices and strands (Table 2; Fig. 3l), the ME-residues in 3ddu have more in the coils (Table 3). Fifth, overall, ME-residue's energy contribution to 5t88 is much higher than that of 3ddu (~ − 88 kJ/mol) (Tables 2, 3). Sixth, interestingly, since proteins contain multiple salt-bridges, unique ME-residues act as ME-residues for different salt-bridges. Therefore, the times used (TU) of ME-residue is much higher than its unique frequency (Tables 2, 3). The occurrence of this repeated use of ME-residues seems to be an important event in protein folding and stability.

### Homologous microenvironment residues substitution and in silico mutation

**Figure 4:**
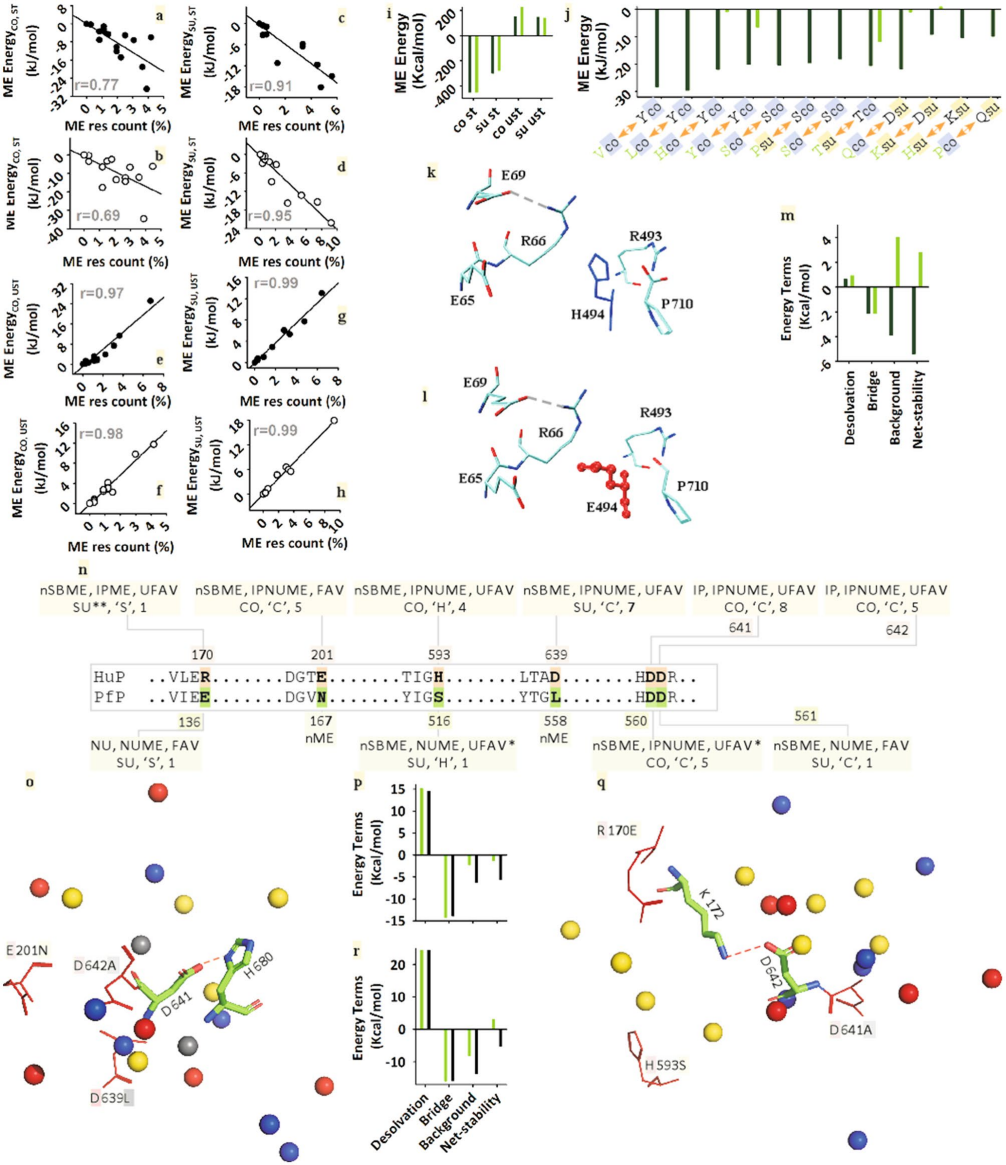
Distribution at the core and surface of 5t88 and 3ddu's ME-residues, and their correlation, interaction-energy, and mutagenesis. 3ddu's (black circle) core's stable (CO, ST) (**a**), surface's stable (SU, ST) (**c**), core's unstable (CO, UST) (**e**), surface's unstable (**g**) (SU, UST) ME-candidates are compared with 5t88's (empty circle) core's stable (**b**), surface's stable (**d**), core's unstable (**f**), surface's unstable (**h**) ME-candidates, respectively. Comparison of 5t88 (black bar) and 3ddu's (green bar)) core (CO) and surface (SU) stable (ST) and unstable (UST) ME-candidates (**i**). Especially in selected ME-candidates of core (blue) and some surfaces (yellow), which are derived from homologous substitution in sequence and, which are favorable to 5t88 (black bar) in respect of 3ddu (green bar) (**j**). Salt-bridge and ME-residues of Wild (**k**) and Mutant (**l**) 3ddu protein where H494 (blue) was mutated by E494 (red). Wild (green bar) and mutant (black bar) 3ddu's components and net energy terms (**m**). Compared to PfP, some of HuP's homologous unfavorable ME-residues and their binary details (**n**). Acidic (red), basic (blue), polar (yellow), and hydrophobic (grey) ME-residues of the salt-bridge, D641-H680 (**o**). Here, the residues that are used for the cumulative replacements are shown by redsticks. The energy terms of the mutant (black) and wild-type HuP (**p**). A similar presentation of ME residues (**q**) and energy terms (**r**) are shown for the salt-bridge, K172-D642.

The net stability of 5t88 is − 78.2 kcal/mol more favorable than that of 3ddu. As the count and strength of salt-bridges have increased, so has the contribution of residue-specific ME-energy. ME-energy is ~ − 90 kJ/mol more favorable in 5t88 than that of 3ddu. How did 5t88 achieve the latter? Favorable and unfavorable ME-residues are located at the core and surface of both proteins (Fig. 4a–h). In all these favorable or unfavorable cases, the count of ME-candidates is highly correlated with their ME-energy. Notably, in the case of 5t88, the contribution of the core's favorable ME-population (CO, ST) is equivalent to that of 3ddu (difference ~ 1.0 kJ/mol), but in the case of the surface (SU, ST), it is greater (difference ~ − 20 kJ/mol) than that of the latter (Fig. 4a–d,i). Again, although unfavorable ME-energy is slightly higher (8.0 kJ/mol) in the case of core (CO, UST) (Fig. 4e,f,i), due to the low unfavorable ME-population at the surface (SU, UST), the contribution of the energy of 5t88 is − 78.0 kJ/mol as compared to 3ddu (Fig. 4g–i). This means that just as the unfavorable ME-population has been removed from the core, so has the favorable ME-population recruited at the surface.

Few cases have been presented (Fig. 4j) to check the ME-energy gain in 5t88 relative to 3ddu due to homologous substitution (Supplementary Figure S1) in sequence and change of location (core and surface). In most cases, there is a ME-energy gain in 5t88 for the amino acid substitution and location change i.e. core (blue) to surface (yellow) transition or vice versa relative to 3ddu's ME-residue (green). Besides, the energy gain is visible in 5t88 even though the location and substitution remain silent. Inspired by this intrinsic method of ME-energy gain in thermophilic 5t88, we examine the effect of substitution of an unfavorable ME-residue (H494E) of 3ddu by the in silico method (Fig. 4k,l). It is noteworthy that while the background and net energy of the salt-bridge in wild-type proteins are unfavorable, in mutant proteins it is highly favorable (Fig. 4m).

In protein thermostability, the importance of specific homologous charged and polar residues other than salt-bridges is immense^12,27–29^. Here, we use the intrinsic sequence property of PfP to increase the thermostability of HuP (Fig. 4n–r). HuP's D641 and D642 residues are conserved like PfP's. Unlike PfP, in HuP, in addition to the microenvironment, these residues also participate in the IP types of salt-bridge (Fig. 4n). Notably, they are highly unfavorable as microenvironmental residues (Fig. 4n; Supplementary Table S13). Again due to unfavorable effects of some microenvironment residues, the net-stability of the salt-bridge, K172-D642 (highly unfavorable ME-residues, R170, H593, D641) is unfavorable, and that for H680-D641 (highly unfavorable ME-residues, E201, D639, D642) is only marginally stable. This means that these two residues act as unfavorable ME-residues in each other's microenvironment. In the case of PfP, these two residues (i.e. D560 and D561) do not participate in the salt-bridge. The one with the lowest repetition i.e. D561 is surface directed (Fig. 4n). Again, even though R560 is unfavorable, it is 6–7 times less than that of D641 (Fig. 4n; Supplementary Table S12 and S13). In each of these two salt-bridges, what are the individual and cumulative effects of the most unfavorable ME-residues (mentioned above) compared to PfP? These results are shown in Fig. 4o–r. Replacements of the homologous ME-residues of HuP are done as they are in the PfP sequence (Fig. 4n). Favorable outcomes are obtained for individual replacements (data not shown), which are 8–14 times higher in the case of cumulative mutations (Fig. 4p,r). Since these residues are used repeated time on both salt-bridge and ME, we looked at the global effect of cumulative mutants. The overall electrostatic stability of these cumulatively mutant proteins is seen to be higher than the wild-type HuP.

## Discussion

Unlike mesophiles, thermophiles operate their biochemical machinery at high temperatures. Since the codes in the sequence are the source of the weak force^7^, and since the three-dimensional structure has both favorable and unfavorable forms of the weak force, the protein structures are marginally stable and functionally interactive^8,9^. Surprisingly, the conformational stability of proteins is almost the same in mesophiles and thermophiles^8,9^. To achieve the state of function, many changes are observed in thermophilic proteins such as reduction of loops^13^, lower non-polar surface^4,18^, high helix content^14,49^ bias on specific amino acids^11,12^ and modulation of weak forces like hydrogen bonds^12^ hydrophobic^4,15–17^ salt-bridge^3,12,19–25^ and van der Waals force^50^. Since the above-mentioned favorable characteristics and weak forces are originated from the amino acid sequence and since there are many variations in the homologous position in different sequences, a general strategy of protein thermostability is unlikely. Compared to HuP, the number of loops in PfP has decreased by 94 residues and the number of ion-pairs, in turn, has increased^19,24,33^. Incidentally, the energy and binary properties of these ion-pairs and their microenvironments are unknown today. We have tried to understand the protein thermostability of the former through a comparative study of PfP and HuP on the salt-bridge and its microenvironment using the PBE based solver^45^, PDB2PQR^51^, IPM^36–39^, and NUM^40^. Here, it has to be kept in mind that the PBE is an approximate procedure to assess atomic potential and thus, the electrostatic energy of salt-bridge. In this computation, the effect of the hydrophobic and other similar non-Coulombic forces is ignored. We used the 0.2 M NaCl to avoid high charge density. All other input parameters were as default or as intermediate level (Supplementary Table 6).

The electrostatic stability of 5t88 is − 78.2 kcal/mol higher than that of 3ddu. In order to increase the frequency of salt-bridges, which exists in 5t88, it is necessary to increase the number of acidic and basic partners of the salt-bridge. This is the reason for the high hydrophilicity of the sequence of the former. For the same reason, it seems that the frequency of a certain type of polar residue is less frequent so that the partners of salt-bridge can be accommodated in the sequence space of 5t88. Relative to 3ddu, such changes in intrinsic properties, such as an increase in the hydrophilicity of the sequence of 5t88, are due to an increase in the number of salt-bridges. It has been shown earlier that the number of salt-bridges in 5t88 is higher^19,24,33^. Our current work shows that the above observation is not the only reason for 5t88's thermostability. Surprisingly, the electrostatic strength of each salt-bridge of 5t88 is greater than that of 3ddu. Salt-bridge strength increases depending on its partners' spatial orientation, geometry, location in protein (core and surface), partner's pair composition, and microenvironment. Our study here shows that the above factors are especially advantageous in the case of 5t88 compared to 3ddu.

Desolvation is an energetically costly process. Again, if the partners of the salt-bridge are in the core, the desolvation energy will be more^36–39,41^. Since most of 5t88's salt-bridges are at the surface (67%), the desolvation cost will be lower than that of 3ddu (58%). The difference between the homologous positions of PfP and HuP is about 75%^24^. In the case of the former, most of these substituted residues are hydrophilic, facilitating the salt-bridge to move towards the surface. Since the surface of the protein has a higher dielectric constant than the core, it is hoped that the bridge energy will be less favorable^37^. Instead, 5t88's bridge-energy is higher than that of the 3ddu, indicating a novel intrinsic strategy. Of the six possible pairs of salt-bridge (i.e. RE, RD, KE, KD, HE, HD), in 5t88, the RE and KE pairs (and RD to some extent) are more than that of 3ddu. The sum of the frequency of HD and HE pairs is almost negligible. Since the salt-bridges made by RE and KE have a special conformational advantage^47^ their abundance in 5t88 seems to be related to its strength. Also, these pairs promote the helix structure as their partners have high helical propensities^48^. The order structure of the helix may also promote the symmetric orientation of the salt-bridge partners, which, in turn, may increase the strength of the salt-bridge. Thus, the relative increase in these residues increases the strength of the salt-bridge as well as the helix stability of 5t88^12,14,19–25^.

When desolvation and bridge energy terms depend only on salt-bridge residues, background energy term, on the other hand, depends on residues of the protein other than salt-bridge residues. Applying an energy cut-off at a very low level (± 0.75 kJ/mol) shows that only about fifteen or fewer residues per salt-bridge act as ME-residues. ME-residue of 5t88 and 3ddu obtained in this way compares ME-energy, residue-class, secondary structure type, and location properties.

ME-residue itself can be an IP or NU partner or non-salt-bridge candidate. An IP or NU partner (acidic or basic) of one salt-bridge may act as ME-candidate in another IP or NU or both types of salt-bridge. Non-salt-bridge charged, polar, PG, hydrophobic residues also participate as microenvironment residue. For all of these reasons, the microenvironment's interactions are very intricate, repetitive, and interlinked across a wide 3Dspace of proteins. Since ME-residues are oriented around the positive and negative partners of salt-bridge, ME-energy is equally likely to be favorable, or unfavorable, or neutral. Despite these possibilities, 5t88's ME-energy is ~ − 90.0 kJ/mol more favorable than the 3ddu. There are many reasons for this energy advantage in the former. In 5t88, compared to the 3ddu, the ME-candidates are more inclined towards order structures like helix and strand. Similarly, just as salt-bridges are surface-directed, so are 5t88's ME-residues surface-directed. Further, relative to 3ddu, 5t88 makes more overlapping and exhaustive usage of ME-candidates. This means that just as a unique non-salt-bridge residue is used as a ME-residue for various salt-bridges, so is a salt-bridge partner used as ME-residue for various other salt-bridges. This phenomenon seems to play a significant role in protein thermostability and protein folding due to a large number of repetitions of a small number of unique ME, which can be seen in 5t88 (and in 3ddu to some lower extent). This strategy seems to be novel as the size of the 5t88 is smaller than that of the 3ddu as the loop parts are removed for thermostability^19,33^ For the same reason, some amino acids (such as Ser, Tyr, etc.) are less abundant in the sequence, but their usage and energy contribution as ME in 5t88 is much higher than in 3ddu. At the core and surface of 5t88, many homologous substitutions occur in favor of these amino acids for the enrichment of its ME. Specifically, relative to 3ddu in 5t88, the core residues (Val, Leu, etc.) in which the ME's contribution is negligible are exactly the ones that are substituted by polar residues (Tyr, Ser, etc.). In many cases, 5t88 enjoys ME-energy advantage as these substituted residues have transitioned from the surface to the core and vice versa. Again, the location and homologous position remain the same, but 5t88's ME-energy is favorable. This seems to be possible with the relocation of the salt-bridge or the recruitment of a new salt-bridge. In this way, 5t88 replaces the homologous amino acid in its sequence relative to 3ddu, making the unfavorable ME-residue a favorable one. In this sense, the homologous amino acids of 3ddu that are acting as favorable ME-residues are almost unchanged. The presence of favorable and unfavorable ME-residues in the core and surface of both proteins seems to be due to the maintenance of the characteristic balance between flexibility and rigidity^8,9,28^. Again, amino acids that are thermolabile (e.g. Gln, Asn, etc.) are almost invisible in 5t88's ME^11^. However, it seems that the reason why the ME-population of 5t88 is composed of less unfavorable and more favorable compared to 3ddu is to enhance the thermostability of the former. In addition to incorporating polar residues into the core of 5t88, other mechanisms operate to stabilize it^15–18^. In this sense, the surface region is relatively more obscure, and thus, surface-directed salt-bridges in 5t88 play a significant role in thermostability.

Protein engineering is important for understanding the basic mechanism of protein function as well as its practical applications^26–30,52^. It is important to gain knowledge about the thermostability of 5t88 in comparison to its mesophilic counterpart as it has a vital role in metabolism and wide application in biotechnology and therapeutics^17,31–33^. In this context, the major problem is which residue will be used as the target residue. In the case of salt-bridge, the partners of salt-bridge are usually chosen as the target residue. Our current study demonstrates that ME's residue-specific interaction energy and other binary details will be useful for this task. Replacing unfavorable residues in ME's population and inspecting its effects seems to be the first step in protein engineering. It is possible to increase the stability of proteins by mutating one or more target ME-residues^27–29^. The stability of the salt-bridge of HuP can be increased by replacing ME-residues with the ones that are present in PfP's homologous positions. This may indicate that the arrangement of amino acids in the sequence of PfP naturally imparts thermostability. It needs to be mentioned here that, in order to have a prominent global effect, ME-population needs to have the most adverse candidates. Compared to other studies^53^ HuP's ME-population does not have such a candidate in it, and thus, the global effect, although favorable, is less prominent. Although the replacement of our ME-residue stabilizes the unstable salt-bridge, the limitations of the in-silico method need to be kept in mind here. Molecular dynamic simulations and genetic engineering methods will be relevant in this context. Overall, our comparative study also applies to other systems.

## Conclusion

In this work, we have compared the intrinsic sequence property with the structure's salt-bridge and ME-energetics, and other binary properties to understand the thermostability of 5t88 relative to 3ddu. The higher hydrophilicity of 5t88 than that of 3ddu is due to the charged but not the polar residue whose primary purpose is to increase the salt-bridge. As the total count of salt-bridge is more in 5t88 than that of 3ddu, the total energy of the salt-bridge has increased and the electrostatic strength of each salt-bridge has also increased. The strategies that 5t88 has adopted for the latter event are described in this work. To reduce costly-dissolving energy, 5t88 has moved its salt-bridges towards the surface. Increased sequence polarity in PfP has helped in this design. To increase favorable bridge energy's strength, PfP has recruited pairs of salt-bridge that have more helical propensity and energetically advantageous. Apart from these, PfP has made a radical change in its ME-residues. Just as PfP has reduced the number of unfavorable ME-candidates compared to HuP's, it has energetically strengthened the microenvironment by means of favorable homologous substitutions. In this regard, massive polar substitutions even at the cost of hydrophobic residues happen in the core of PfP. The study, further, demonstrates that it is possible to increase the stability of a salt-bridge by replacing an unfavorable ME-residue with a favorable one. Thus, ME-energy and binary details seem to be useful for different aspects of protein engineering.

## Methods

### Residue classes and sequence composition

Three broad classes of amino acids are used as devised by Bett and Russel^54^. Salt-bridge forming residues (sbfrs) are constituted by both the acidic (Asp D and Glu E) and basic (Arg R, Lys K, and His H) amino acids. These residues are also polar^54^. However, here we subcategorize these residues into salt-bridge residues (sbfrs). Others are hydrophobic (Cys C, Ala A, Leu L, Met M, Ile I, Val V, Phe, F, Pro P, Gly G) and Polar (Asn N, Gln Q, Ser S, Thr T, Tyr Y, and Trp W).

Sequence alignment was performed using the T-coffee alignment method^44^. Insertion and deletion regions were excised and a sequence block was prepared. Sequence composition was analyzed using the unblock and block format of sequence^55^. Hopp and Woods hydrophilicity^43^ was analyzed using the block format of the sequence of 5t88 and 3ddu.

### Side-chain accessibility and average accessibility

The relative accessibility (%) of the side chain of salt-bridge residue (acidic D and E, and basic R, K, and H) for isolated and network types and ME-residues of salt-bridges of a given protein (5t88 or 3ddu) were obtained using the following formula.

$$ASA_{SCrel} = \frac{{ASA_{SCfolded} *100}}{{ASA_{SCunfolded} }}$$

*ASA*_*SCfolded*_ is the sum of absolute atomic ASA of the side-chain (SC), except for the main-chain (Cα, C, N, O), for a given residue in the folded state of a protein. It is obtained by the implementation of the program, Surface Racer^56^ or NACCESS^57^. In the unfolded state, *ASA*_*SCunfolded*_ for the residue was procured from the table value^58^. These values were used in the above formula to obtain the relative (%) ASA (*ASA*_*SCrel*_) for the residue. Average *ASA*_*SCrel*_ for isolated type (IP) of salt-bridge is the sum of *ASA*_*SCrel*_ for acidic and basic partners divided by two. For network type (NU), where ≥ 2 acidic or basic partners are linked with a basic or an acidic residue respectively, the sum of *ASA*_*SCrel*_ for all unique partners is divided by the total count of unique partners of the NU for obtaining the average *ASA*_*SCrel*_. Average *ASA*_*SCrel*_ for IP and NU types of salt-bridges was computed at the time of the extraction of IP and NU types of salt-bridges from a protein by an automated procedure^59,60^. Similarly, *ASA*_*SCrel*_ values for ME-residues were also extracted for their subsequent use.

### Energy terms for isolated and network pairs

An IP of salt-bridge is formed by the side-chains of an acidic (D or E) and a basic (H or R or K) residues within 4 Å distance. On the other hand, a NU is formed when more than one acidic or basic residue is interacting with a basic (base-net) or an acidic (acid-net) residue respectively within a 4 Å distance. When base-net and acid-net are connected, a mixed NU is formed^38^. While IPM was suggested for IP^36–39^ for NU, NUM was used for the computation of the component (*ΔΔG*_*dslv*_, *ΔΔG*_*brd,*_ and *ΔΔG*_*bac*_) and net (*ΔΔG*_*net*_) energy terms^40^. 3ddu and 5t88 were minimized using AUTOMINv1.0^61^. IP and NU types of salt-bridges of these proteins were extracted by the use of a modified version of our earlier program^59,60^. PDB2PQR v1.9.0^51^ was used to generate the partial atomic charge (Q) and radius (R) of the PDB files using the parameters of the force field, CHARMM22. Based on the model and component terms, the initial PDB2PQR file of the PDB file^51^ was subjected for mutation main chain of the partners of salt-bridge and main-chain and side-chain of other residues of the protein by the use of hydrophobic isosteres. For each IP salt-bridge, five mutated structures were prepared to obtain the component energy terms, of which three were in the folded, and two were in the unfolded states. While one of the folded states contains the side-chain of both the partners, the other two contain the side-chain of either of the partners (acidic or basic) of the salt-bridge. In unfolded states, only the side-chain of either of the partners (acidic or basic) of salt-bridge was present along with the main-chain of the immediately preceding and following residues^37^. The rest of the residues of the protein were absent in these states. While the computation of desolvation energy involves both the folded and unfolded states^37–39^ bridge and background energy were computed using only the folded states as earlier^36–39^. We performed four states computation on each of the acidic and basic partners to obtain their desolvation energy separately^62–64^. Thus, for a given salt-bridge, preparation of five different mutated PDB structures and nine different APBS runs were required to obtain the component energy terms^39,63–65^. A higher version of the earlier programs^63–65^ was used for automated extraction of the component and hence the net energy terms^53^.

Unless stated otherwise, the computation of atomic potentials was performed using the manually configured multigrid and linearized Poisson-Boltzmann calculation with a single Debay-Hückel boundary condition by using APBSv1.3^45^. Notably, the iterative focus boundary condition, which was found to give a minor improvement of the outcome^38,39^ was avoided as it involves lengthy computation time for larger proteins like ours. For all computations, the grid-center was set at a protein-specific center for both the folded and unfolded state of the protein. It was (3.559, 12.327, 33.122) for 3ddu_A and (134.810, 17.098, 141.146) for 5t88_B. 3D grid dimension that depends on both the grid spacing and dimension of the protein (plus addition 20 Å space in each dimension) was set appropriately to the closest predefined integer values^37^. It was 129 × 97 × 129 for 3ddu and 97 × 97 × 129 for 5t88 for all computations. Mobile ion (univalent) concentration was set to 0.20 M. The dielectric constants of the protein and the solvent were set to 3.5 and 80.0, respectively. Other APBS input parameters (such as temperature, solvent-radius, dielectric and ion-accessibility coefficients, etc.) were kept as default if not stated otherwise. In the case of Histidine containing salt-bridges, a fully protonated form of the imidazole ring (side-chain charge + 1) was used. The main-chain atoms of GLY were N, CA, C, O, H, HA2, and that for the other residues were N, CA, C, O, H, HA. APBS generates the free energy of energy terms in kT unit (k is the Boltzmann constant), which was then multiplied by 0.00199 and the value of temperature to get the energy in kcal/mol unit. Notably, the more the value of set temperature more will be the value of the free energy. Energy terms (component and net) of salt-bridge and interaction-energy of ME-residues were extracted for the A and B chain of 3ddu and 5t88 respectively using identical run-time parameters of APBS. Post-run analyses were performed using the Microsoft EXCEL and Sigma Plot v12.0 programs.

NUM, unlike IPM that performs pair-wise computation^36–39^ consider all NU-residues of salt-bridge together for the computation of component energy terms. For example, for a NU of one base and two acids, there will be four mutated states, one (combined state) that contains side-chain charges for all the residues and the other three (individual state) with side-chain charges for only one of these residues at a time. Other residues of the protein for these states were mutated by the use of their corresponding hydrophobic isosteres. APBS was run separately on each of these states. Subtraction of electrostatic energy of the latter three individual states from the former combined state would give the *ΔΔG*_*brd*_ for the NU. The electrostatic atomic potential for the combined state was multiplied separately with the side-chain partial charges of all the acidic (except the NU-acid residue), basic (except the NU-base residue), polar and hydrophobic residues of the protein to obtain acidic (A), basic (B), polar (P) and hydrophobic (H) parts of energy, whose sum was taken as *ΔΔG*_*bac*_. In these computations, inclusions of the unfolded states were neglected as earlier^37^. In NUM, *ΔΔG*_*dslv*_ for each of the partners of NU was computed separately in a pair-wise manner involving both the folded and unfolded states^62^ of the protein following the IPM. This way duplicate entries of the *ΔΔG*_*dslv*_ will appear for the common partners of NU, which were then discarded. The sum of the unique entry of each partner of the NU is the *ΔΔG*_*dslv*_.

### Microenvironment analysis

The side chains of the partners of a salt-bridge could be surrounded by isolated charged, polar and hydrophobic residues of a protein. These residues influence the background and hence, the net energy terms of a salt-bridge^36–39,53^. When all salt-bridges of a protein are considered, we obtain a collection of these residues. How each of these residues affects the stability of a protein? A residue is taken as ME of a salt-bridge, when the energy of interaction between the partners of a salt-bridge and the residue is lower than (stabilizing) or higher than (destabilizing) a preset energy cut-off. We set the cut-off at 0.75 kJ/mol^53^. We then raised the following questions. Do the ME-residues of protein act favorably, unfavorably, or neutrally? Is there a preference for ME-residues for a type of secondary structure (helix, strand, or coil)? Is there a preference for ME-residues for a given residue class (charged, polar, or hydrophobic)? How do the ME-residues distribute in the core and surface of a protein? We worked out the interactionenergy and all the above-mentioned questions for the ME-residues of 3ddu and 5t88. Here, the details of the computation of ME-residues for the IP and NU types of salt-bridge were practically different. For IP type, in the folded state, except the side chain of the partners of salt-bridge, other residues of the protein were mutated by the use of hydrophobic isosteres. APBS^45^ was run by keeping all the run-time parameters identical as mentioned above. Atomic potentials thus generated were multiplied by their corresponding atomic partial charges of the side chain and the constant (kTx4.2), i.e. 2.492 (i.e. 0.00199 × 298.15 × 4.2) to obtain the atomic energy in kJ/mol for all the residues of the protein. Here, to exclude the partners of salt-bridge from the ME-population, their atomic partial charges were set to zero prior to the above multiplication. Residue-specific energy was then obtained by adding the energies of its atoms in the side chain. Here, details of residue-specific accessibility, type of secondary structure, residue-class, and core or surface location were also included in the above residue-specific file. These residues were then screened against the energy cut-off i.e. 0.75 kJ/mol. Those residues were taken as ME of the salt-bridge whose interaction-energies were higher than 0.75 kJ/mol (unfavorable) or lower than − 0.75 kJ/mol (favorable). This way ME-residues for all salt-bridges of a protein was extracted, which were taken as the ME of the protein for IP type of salt-bridges. Secondary structure information was extracted from the PDB file. Side-chain accessibility of ME-residues was determined as earlier (see above). Knowledge of residue classes was taken from the literature^54^.

ME-residues for an NU type of salt-bridge of protein were computed in the presence of the side chains of all partners of the NU. For example, for an NU of one base and two acids, a mutated structure file was generated wherein except the side chains of the NU partners, other residues of protein were mutated by their corresponding hydrophobic isosteres. The rest of the computation for obtaining ME of protein for NU types of salt-bridge was similar to IP-type as above. For a protein, two files of ME-residues (one for IP and the other for NU) were obtained along with the details on residue-specific interaction-energy (favorable, unfavorable, and neutral), type of secondary structure (Helix or strand or coil), residue-class (charged or polar or hydrophobic) and core or surface location.

Because a given residue can participate as a ME-residue for multiple salt-bridges or a given charged residue is forming a salt-bridge and behaving as ME-residue for other salt-bridge(s) or a given residue is of overlapping type i.e. a ME-residue for both IP and NU types of salt-bridge, the protein-specific ME-output further processing. All these ME-residues specific details were further extracted and analyzed using the manual procedure by the use of the Excel program.

### In silico mutation

Unfavorable (positive interaction-energy) and favorable (negative interaction-energy) ME-residues at different locations (core or surface) and in a segment of secondary structure (helix or strand or coil) of protein could be identified. Mutation of the targeted ME-residue was performed in Swiss-Pdbviewer, v4.1.0^53,66^ by using the lowest steric clashed (with the main and side chains of neighboring residues) rotamer of the residue. The minimized structure of a protein was used for the targeted mutation for a highly unfavorable or favorable residue. The mutated structure of the protein was saved without further minimization. This mutated structure was used for the evaluation of the energy terms for the targeted salt-bridge for which the ME-residue was acting as the microenvironment.

## Supplementary Information

Supplementary Information 1.

## Data Availability

While the coordinate files, 3ddu and 5t88 are available in the Protein Data Bank, these structure files were minimized for their use in the study. Minimized files are available upon request.

## Abbreviations

PfP: Prolyl oligopeptidase from *Pyrococcus furiosus*
HuP: Prolyl oligopeptidase from human
PBE: Poisson–Boltzmann equation
ME: Microenvironment
APBS: Adaptive Poisson Boltzmann Solver
IP: Isolated pair
NU: Network unit
IPM: Isolated pair method
NUM: Network unit method
IPME: Isolated pair's partner as ME-candidate
NUME: Network unit's partner as ME-candidate
SBnME: Salt-bridge's partner but not ME-candidate
nSBME: ME-candidate but not salt-bridge's partner
nSBnME: Neither ME-candidate nor salt-bridge's partner

## Code availability

Binary version of the fully automated AWK-scripts was used for the analysis of isolated pair and network unit types of salt-bridge by isolated pair method and network unit method, respectively. Scripts are also used for the analysis of the energy terms of the network unit by isolated pair method for its comparison with the NUM-derived results. Scripts are also there to analyze the microenvironment of isolated-pair and network-unit by IPM and NUM, respectively. While we have tested the program to be fully functional, error-free, and user-friendly for the proteins 5t88, 3ddu, and a few others, we are presently continuing our this verification works. The binary version of these could be obtained upon request by experts, working in the field.
