# Supplementary material for: Intrinsic basis of thermostability of prolyl oligopeptidase from Pyrococcus furiosus
Source: Sci Rep. 2021 Jun 2;11:11553. doi: 10.1038/s41598-021-90723-4 (PMC8172842; doi:10.1038/s41598-021-90723-4)
Supplement: Supplementary file 1 — Supplementary Information 1. [file 41598_2021_90723_MOESM1_ESM.doc]

Intrinsic Basis of Thermostability of Prolyl Oligopeptidase from *Pyrococcus furiosus*

Sahini Banerjee ^a, #^, Parth Sarthi Sen Gupta ^b, #^, Rifat Nawaz Ul Islam ^c, #^ and Amal Kumar Bandyopadhyay ^d^, *

**Supplementary Table 1 | Aligned sequence of the Prolyl oligopeptidase from *Pyrococcus furiosus* (PfP, 5t88) and human (HuP, 3ddu) used for evaluation of sequence hydrophilicity**. The sequence of 3duu_A (710) and 5t88_B (616) were procured from the UniProt database and aligned using the T-COFFEE program, Version_11.00.d625267. The insertion regions were removed manually from both these sequences. The remaining 616 positions are analyzed for sequence property using Hopp and Woods's (1981) hydrophilicity index for amino acids by the use of the program PHYSICO2. The window size was 9.0. The raw data thus obtained were smoothed using SIGMAPLOT v12.0 in-built procedure with the negative exponential as smooth function with sampling proportion two and polynomial of degree two.

>3ddu_A_ UniProtID_P48147_EC_3.4.21.26;

ICDPYAWLEDPDSEQTKAFVEAQNKITVPFLEQIRGLYKERMTELYDYPKYSCHFKKGKRYFYFYNTGLQDDGTVALRGYAFSEDGEYFAYGLSASGSDWVTIKFMKVDGAKELPDVLERVKFSCMAWTHDGKGMFYNSYPQQDGKSDGTETSTNLHQKLYYHVLGTDQSEDILCAEFPDEPKWMGGAELSDDGRYVLLSIREGCDPVNRLWYCDLQQESSGIAGILKWVKLIDNFEGEYDYVTNEGTVFTFKTNRQSPNYRVINIDSKWKVLVPEHEKDVLEWIACVRNFLVLCYLHDVKNILQLHDLTTGALLKTFPLDVGSIVGYSGQKKEIFYQFTSFLSPGIIYHCEPRVFREVVKIDASDYQTVQIFYPSKDGTKIPMFIVHKKDGSHPAFLYGYGGFNISITPNYSVSRLIFVRMGGILAVANIRGGGEYGETWHKGGILANKQNCFDDFQCAAEYLIKEGYRLTINGGSNGGLLVAACANQRPDLFGCVIAQVGVMDMLKFHKYTIGHAWTTDYGCSDSKQHFEWLVKYSPLHNVKLPIQYPSMLLLTADHD**D**RVVPLHSLKFIATLQYIVGPLLIHVDTKAGHGGKPTAKVIEEVSDMFAFIARCLN

>5t88_B_ UniProtID_Q51714_EC_3.4.21.26;

MEDPYIWMENLEDERVLKIIEEENKRFREFIGELSDKLFPEVWEQFSQPTIGMARITKKGIIASYSEKDRVVIKWFNGDVIVDSKELEREVGDEVLLQGFTTDEEGEKLAYSFSIGGADEGITRIIDLKTGEVIEEIKPSIWNITFLKDGYYFTRFYRKEKTPDGVNPPAARMFWKDREGERMVFGEGLTSGYFMSIRKSSDGKFAIVTLTYGWNQGEVYIGPIDNPQEWKKVYSASVPVEAIDVVNGKLYILTKEGKGLGKIIAIKNGKIDEVIPEGEFPLEWAVIVRDKILAGRLVHASYKLEVYTLNGEKIKEITFDVPGSLYPLDKDEERVLLRYTSFTIPYRLYEFKDDLRLIEERKVEGEFRVEEDFATSKDGTKVHYFIVKGERDEKRAWVFGYGGFNIALTPMFFPQVIPFLKRGGTFIMANLRGGSEYGEEWHRAGMRENKQNVFDDFIAVLEKRKKEGYKVAAWGRSNGGLLVSATLTQRPDVMDSALIGYPVIDMLRFHKLYIGSVWIPEYGNPEDPKDREFLLKYSPYHNVDPKKKYPPTLIYTGLHDDRVHPAHALKFFMKLKEIGAPVYLRVETKSGHMGASPETRARELTDLLAFVLKTLS


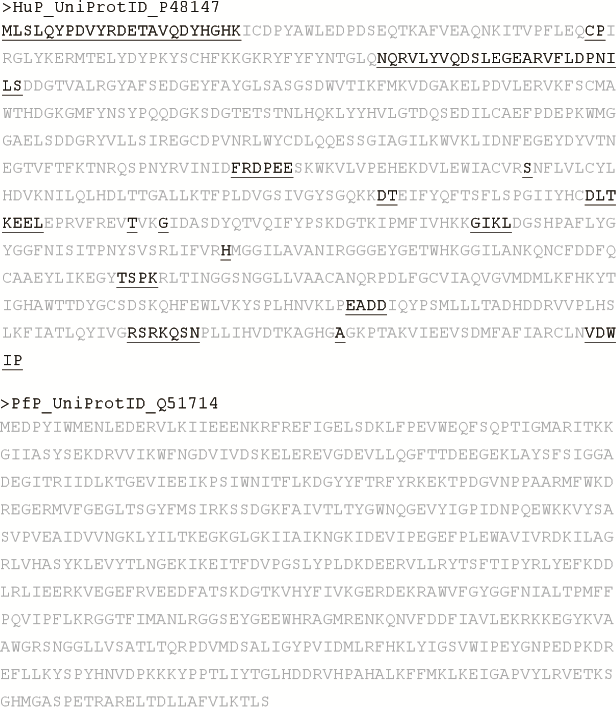


**Supplementary Figure 1 | Alignment of** Q51714 (**5t88) and** P48147 (**3ddu**) **showing restored homologous positions** (grey colored residues i.e. 616 residues). Deleted residues of P48147 are shown as underlined black-colored residues.

**Supplementary Table 2 | Grand average hydrophilicity of 5t88_B is positive and that for the 3ddu_A is negative.** Grand average hydrophilicity of 3ddu_A and 5t88_B was determined using the above-aligned sequence using the program, PHYSICO2. Amino acid index values were taken from Hopp and Woods (1981).

| **Aligned Sequence** | **Grand Av. Hydrophilicity** |
| --- | --- |
| 3ddu_A | -0.11 |
| 5t88_B | 0.16 |

**Supplementary Table 3 Relative abundance of polar residues are lower in 5t88_B than 3ddu_A.** Absolute and normalized (%) frequency of amino acids of 3ddu_A (709) and 5t88_B (616) along with their relative abundance (RA) in the latter in reference to the former. The RA is the difference of % frequency amino acids of 5t88_B and 3ddu_A. sbfrs is the salt-bridge forming residues (D, E, R, H, K).

| **Residue Class** | **Amino acids** | **3ddu_A**  **709** | **5t88_B**  **616** | **[v1]**  **3ddu_A**  **%** | **[v2]**  **5t88_B**  **%** | **Relative abundance**  **[v2]-[v1]** |
| --- | --- | --- | --- | --- | --- | --- |
| sbfrs | E | 41 | 62 | 5.8 | 10.1 | 4.3 |
|  | R | 25 | 35 | 3.5 | 5.7 | 2.2 |
|  | K | 47 | 53 | 6.6 | 8.6 | 2 |
|  | D | 53 | 37 | 7.5 | 6 | -1.5 |
|  | H | 23 | 9 | 3.2 | 1.5 | -1.7 |
| Hydrophobic | C | 16 | 0 | 2.3 | 0 | -2.3 |
|  | A | 36 | 29 | 5.1 | 4.7 | -0.4 |
|  | L | 62 | 50 | 8.7 | 8.1 | -0.6 |
|  | M | 11 | 13 | 1.6 | 2.1 | 0.5 |
|  | I | 40 | 46 | 5.6 | 7.5 | 1.9 |
|  | V | 48 | 43 | 6.8 | 7 | 0.2 |
|  | F | 36 | 34 | 5.1 | 5.5 | 0.4 |
|  | G | 57 | 53 | 8 | 8.6 | 0.6 |
|  | P | 31 | 30 | 4.4 | 4.9 | 0.5 |
| Polar | N | 26 | 17 | 3.7 | 2.8 | -0.9 |
|  | Q | 27 | 8 | 3.8 | 1.3 | -2.5 |
|  | S | 40 | 27 | 5.6 | 4.4 | -1.2 |
|  | Y | 41 | 29 | 5.8 | 4.7 | -1.1 |
|  | T | 37 | 29 | 5.2 | 4.7 | -0.5 |
|  | W | 12 | 12 | 1.7 | 1.9 | 0.2 |

**Supplementary Table 4 | Relative abundance of R, K, and E are higher in isolated pair and network unit types of salt-bridges of pfPOP in reference to huPOP.** Total 42 and 56 acidic (E and D) and basic (R, K, and H) residues are participating in the formation of 21 and 28 isolated pairs (IP) in 3ddu and 5t88 respectively. Similarly, 41 and 53 acidic (E and D) and basic (R, K, and H) residues are participating in the formation of 30 (12 network units) and 37 (16 network units) network pairs (NU) in 3ddu and 5t88 respectively. Absolute and normalized compositions for these five residues are shown along with the relative abundance for these residues (RA_sb) for IP and NU types of salt-bridges respectively.

| Salt-bridge forming residues  (sbfrs) | Absolute count | | | | Normalized (%) count | | | | Relative  abundance | |
| --- | --- | --- | --- | --- | --- | --- | --- | --- | --- | --- |
|  | 3ddu_IP | 3ddu_NU | 5t88_PI | 5t88_NU | [a] %  3ddu_IP | [b] %  3ddu_NU | [c] %  5t88_PI | [d] %  5t88_NU | [a-c]  RA sb_PI% | [b-d]  RA sb_NU% |
| K | 10 | 7 | 16 | 11 | 23.8 | 17.1 | 28.6 | 20.8 | 4.8 | 3.7 |
| R | 6 | 8 | 11 | 12 | 14.3 | 19.5 | 19.6 | 22.6 | 5.4 | 3.1 |
| H | 5 | 4 | 1 | 2 | 11.9 | 9.8 | 1.8 | 3.8 | -10.1 | -6.0 |
| D | 14 | 13 | 11 | 11 | 33.3 | 31.7 | 19.6 | 20.8 | -13.7 | -11.0 |
| E | 7 | 9 | 17 | 17 | 16.7 | 22.0 | 30.4 | 32.1 | 13.7 | 10.1 |
| Total | 42 | 41 | 56 | 53 | 100 | 100 | 100 | 100 | 0 | 0 |

**Supplementary Table 5 | RE and KE are abundant in network-unit (NU) and isolated-pair (IP) types of salt-bridge in PfP.** Absolute (abs) count of six possible pairs (RE, KE, RD, KD, HE, and HD) in NU and IP types of salt-bridges are normalized in % scale with respect to the total of these types. At the same time, the absolute count of total (sum of NU and IP types) pair-types of salt-bridge is also normalized similarly. Relative abundance (RA) is the difference of the normalized value of a type (NU, IP, or Total) of PfP in reference to that of HuP.

| **Pair** | **NU (abs)** | | **IP (abs)** | | **NU (%)** | | **IP (%)** | | **RA (%)** | | **Total (abs)** | | **Total (%)** | | **RA (%)** |
| --- | --- | --- | --- | --- | --- | --- | --- | --- | --- | --- | --- | --- | --- | --- | --- |
|  | **5t88** | **3ddu** | **5t88** | **3ddu** | **5t88** | **3ddu** | **5t88** | **3ddu** | **NU** | **IP** | **5t88** | **3ddu** | **5t88** | **3ddu** | **total** |
| RE | 10 | 6 | 6 | 3 | 27.0 | 20.0 | 21.4 | 14.3 | 7.0 | 7.1 | 16 | 9 | 24.6 | 17.6 | 7.0 |
| KE | 11 | 4 | 10 | 3 | 29.7 | 13.3 | 35.7 | 14.3 | 16.4 | 21.4 | 21 | 7 | 32.3 | 13.7 | 18.6 |
| RD | 8 | 8 | 5 | 3 | 21.6 | 26.7 | 17.9 | 14.3 | -5.0 | 3.6 | 13 | 11 | 20.0 | 21.6 | -1.6 |
| KD | 5 | 6 | 6 | 7 | 13.5 | 20.0 | 21.4 | 33.3 | -6.5 | -11.9 | 11 | 13 | 16.9 | 25.5 | -8.6 |
| HE | 3 | 3 | 1 | 1 | 8.1 | 10.0 | 3.6 | 4.8 | -1.9 | -1.2 | 4 | 4 | 6.2 | 7.8 | -1.7 |
| HD | 0 | 3 | 0 | 4 | 0.0 | 10.0 | 0.0 | 19.0 | -10.0 | -19.0 | 0 | 7 | 0.0 | 13.7 | -13.7 |
| Total | 37 | 30 | 28 | 21 | 100 | 100 | 100 | 100 | 0.0 | 0.0 | 65 | 51 | 100 | 100 | 0.0 |

**Supplementary Table 6 Run-time parameters of the Adaptive Poisson-Boltzmann Solver (APBS) for the protein 3ddu_A (707) and 5t88_B (616).** The protein was minimized for 1000 steps using AUTOMINZv1.0 prior to the evaluation of the component and net energy terms. Other required parameters of APBS were used as default, in not stated otherwise. These parameters were kept identical for both isolated pair and network unit types of salt bridges. Except for the grid-points and grid-center, other set parameters were identical for 3ddu_A and 5t88_B.

| **protein** | **3ddu_A**  **or**  **5t88_B** |
| --- | --- |
| Temperature | 298.15 |
| Ionic-strength | 0.2 |
| Grid-spacing | 0.83 |
| Accessibility | NACCESS |
| Model for main-chain and side-chain | As earlier |
| Main-chain | Gly:  N,CA,C,O,H,HA2  Other residues:  N,CA,C,O,H,HA |
| HISTIDINE charged state | Fully protonated form of the imidazole ring |
| protein's dielectric constant | 3.5 |
| Solvent dielectric constant | 80 |
| Salt-bridge distance | 4 |
| Force field | CHARMM |
| Microenvironment's energy cut-off | 0.8 kJ/mol |
| Grid-points | 129 x 97 x 129 for 3ddu_A  and  97 x 97 x 129 for 5t88 |
| center of the grid (x, y, z) | 3.6, 12.3, 33.1 for 3ddu  and  134.8 17.1 141.1 for 5t88 |

**Supplementary Table 7 | Average component energy terms are more favorable in PfP (5t88) than HuP (3ddu).** Overall average energy for the component (desolvation ΔΔGdslv, bridge ΔΔGbrd, background ΔΔGbac) and net (ΔΔGnet) energy terms for isolated pair (IP) and network unit (NU) types of salt-bridge (SB) of 3ddu (707 residues) and 5t88 (616 residues). As the coordinate information for 426T and 429G are not available in 3ddu, 707 residues were considered for normalization purposes. The Sum of the desolvation and bridge energies are shown as these two energy terms originate due to the partners of salt-bridge.

| Protein (residues) | Items | Energy terms (Kcal/mol) | | | | |
| --- | --- | --- | --- | --- | --- | --- |
|  |  | I  *ΔΔG_dslv_* | II  *ΔΔG_brd_* | I+II  ΔΔG_dslv_+ ΔΔG_brd_ | III  *ΔΔG_bac_* | I+II+III  *ΔΔG_net_* |
| 3ddu (707) | IP (21 pairs) | 137.10 | -162.35 | -25.25 | -36.37 | -61.62 |
|  | NU (12 NU i.e. 30pairs) | 202.54 | -263.42 | -60.88 | -56.83 | -117.71 |
| 3ddu (100) | IP | 19.39 | -22.96 | -3.57 | -5.14 | -8.72 |
|  | NU | 28.65 | -37.26 | -8.61 | -8.04 | -16.65 |
| 5t88 (616) | IP (28) | 205.81 | -257.85 | -52.04 | -45.80 | -97.84 |
|  | NU (16 NU i.e. 37 pairs) | 196.21 | -287.49 | -91.28 | -66.54 | -157.82 |
| 5t88 (100) | IP | 33.41 | -41.86 | -8.45 | -7.44 | -15.88 |
|  | NU | 29.67 | -46.67 | -14.82 | -10.79 | -25.62 |

**Supplementary Table 8 | Component and net energy terms, bond-multiplicity, average-distance, and accessibility of isolated pair type (21 pairs) of salt-bridge of 3ddu_A (707 residues).** Energy terms are extracted following the isolated pair method (IPM)^35-38^. Background energy is sub-divided into acidic (A), basic (B), and polar (P) parts. The net energy of the salt-bridge is the sum of the component (desolvation, bridge, and background) energy terms. Accessibility of acidic (A) and basic (B) residues were extracted by NACCESS program whose average is the *ASA_av_*.

| Isolated pair (IP)  Type of  Salt-Bridge  (3ddu_A) | *ΔΔG_dslv_* | *ΔΔGbac_A* | *ΔΔGbac_B* | *ΔΔGbac_P* | *ΔΔGbac_H* | *ΔΔG_brd_* | *ΔΔGnet* | Average  distance  (Å) | *ASA_av_* Å^2^ | *ASA-B* Å^2^ | *ASA-A* Å^2^ |
| --- | --- | --- | --- | --- | --- | --- | --- | --- | --- | --- | --- |
| LYS688_ASP675 | 5.94 | 1.54 | -2.41 | 0.35 | 0.17 | -11.52 | -5.93 | 2.77 | 25.7 | 21.2 | 30.2 |
| HIS355_ASP336 | 3.07 | 0.09 | -0.46 | -0.07 | 0.04 | -5.42 | -2.75 | 3.35 | 40.2 | 49 | 31.5 |
| ARG66_GLU69 | 0.9 | 0 | 4.22 | 0.06 | -0.29 | -2.13 | 2.76 | 2.99 | 46.2 | 37.2 | 55.2 |
| ARG260_ASP284 | 7.84 | -1.38 | -0.55 | -1.84 | -0.7 | -8.78 | -5.41 | 3.13 | 28.9 | 23.1 | 34.8 |
| ARG245_ASP265 | 9.12 | 0.52 | -0.42 | -0.04 | -0.03 | -13.7 | -4.55 | 3.13 | 29.6 | 46.2 | 13 |
| LYS325_ASP320 | 4.12 | 0.39 | -0.22 | -0.16 | -0.14 | -7.96 | -3.97 | 2.90 | 46.9 | 46.5 | 47.3 |
| HIS466_ASP463 | 7.67 | -0.66 | 2.2 | -0.89 | 0.56 | -3.89 | 4.99 | 3.59 | 32.3 | 18.1 | 46.5 |
| LYS677_ASP122 | 7.27 | 0.82 | -0.91 | -3.17 | -0.24 | -10.42 | -6.65 | 2.91 | 43.4 | 49.8 | 37 |
| HIS409_GLU393 | 6.94 | 1.28 | -1.09 | -4.44 | -0.9 | -7.66 | -5.87 | 2.72 | 18.4 | 21.3 | 15.5 |
| HIS680_ASP641 | 15.11 | 7.56 | -6.55 | -2.27 | -0.97 | -14.15 | -1.27 | 3.17 | 11.3 | 22.6 | 0 |
| LYS23_ASP18 | 5.24 | 0.38 | -1.44 | -0.15 | 0.01 | -5.85 | -1.81 | 2.48 | 51.7 | 62.2 | 41.1 |
| ARG11_GLU13 | 6.44 | -0.78 | 1.63 | -2.12 | 0.21 | -9.1 | -3.72 | 3.21 | 41.5 | 22.4 | 60.7 |
| ARG111_ASP104 | 5.61 | -0.03 | -1.12 | -0.16 | 0.14 | -5.2 | -0.76 | 3.63 | 57.8 | 54.3 | 61.4 |
| LYS335_ASP356 | 5.03 | 1.04 | -2.22 | -2.55 | -0.3 | -6.4 | -5.4 | 2.83 | 37.5 | 54.8 | 20.1 |
| LYS684_GLU692 | 4.14 | -2.4 | 1.14 | -2.53 | 0.42 | -9.21 | -8.44 | 2.54 | 18.1 | 14.6 | 21.7 |
| LYS48_GLU44 | 1.14 | 0.6 | -0.51 | -0.05 | 0 | -2.53 | -1.35 | 3.02 | 67 | 67.9 | 66.1 |
| LYS172_ASP642 | 24.36 | 5.25 | -4.02 | -9.02 | -0.34 | -15.85 | 0.38 | 3.07 | 0.8 | 0.2 | 1.4 |
| HIS587_ASP603 | 4.09 | -4.81 | 7.05 | 1.36 | 0 | -2.94 | 4.75 | 3.32 | 43.1 | 16.2 | 70 |
| ARG420_GLU418 | 2.97 | 0.4 | -0.02 | 0 | -0.05 | -7.36 | -4.06 | 2.53 | 60.3 | 75.8 | 44.9 |
| LYS445_ASP528 | 2.77 | 3.15 | -2.55 | -4.34 | -0.21 | -2.28 | -3.46 | 3.89 | 45.4 | 73.2 | 17.5 |
| LYS389_GLU296 | 7.33 | -5.06 | 1.61 | -2.79 | -0.19 | -10 | -9.1 | 3.19 | 39.9 | 19.6 | 60.1 |

**Supplementary Table 9 | Component and net energy terms, bond-multiplicity, average-distance, and accessibility of isolated pair type (28 pairs) of salt-bridge of 5t88_B (616 residues).** Energy terms are extracted following the isolated pair method (IPM)^35-38^. Background energy is sub-divided into acidic (A), basic (B), and polar (P) parts. The net energy of the salt-bridge is the sum of the component (desolvation, bridge, and background) energy terms. Accessibility of acidic (A) and basic (B) residues were extracted by NACCESS program whose average is the *ASA_av_*.

| Isolated pair (IP)  Type of  Salt-Bridge  (5t88_B) | *ΔΔG_dslv_* | *ΔΔGbac_A* | *ΔΔGbac_B* | *ΔΔGbac_P* | *ΔΔGbac_H* | *ΔΔG_brd_* | *ΔΔGnet* | Av. Dist  (Å) | *ASA_av_* Å^2^ | *ASA-B* Å^2^ | *ASA-A* Å^2^ |
| --- | --- | --- | --- | --- | --- | --- | --- | --- | --- | --- | --- |
| LYS574_GLU577 | 3.17 | -0.74 | 1.12 | -4.74 | 0.23 | -5.18 | -6.14 | 3.08 | 32.6 | 27.8 | 37.5 |
| ARG55_ASP329 | 5.56 | 2.28 | -1.56 | -0.26 | -0.4 | -5.62 | -7.77E-16 | 3.27 | 24.9 | 28.1 | 21.6 |
| HIS383_GLU371 | 11.72 | -5.76 | 4.72 | -1.69 | -1.52 | -8.23 | -0.76 | 2.84 | 15.9 | 0 | 31.8 |
| ARG296_GLU305 | 15.8 | -0.6 | -0.55 | -4.25 | 0.38 | -17.87 | -7.09 | 3.12 | 3.9 | 4.4 | 3.4 |
| ARG155_GLU181 | 9.26 | -0.44 | 0.72 | 1.26 | -0.26 | -13.66 | -3.12 | 2.98 | 29.3 | 22.7 | 35.9 |
| ARG15_ASP13 | 6.55 | 0.75 | -0.67 | -0.17 | -0.05 | -11.64 | -5.23 | 3.13 | 42.4 | 68.4 | 16.3 |
| ARG70_ASP83 | 10.25 | -2.08 | -0.94 | 0.79 | -0.08 | -7.97 | -0.03 | 3.17 | 19 | 29.8 | 8.2 |
| LYS37_GLU33 | 1.98 | -0.2 | 0.27 | -0.02 | 0.01 | -4.47 | -2.43 | 2.55 | 69.1 | 60.7 | 77.5 |
| LYS547_ASP544 | 5.73 | -0.84 | 0.73 | -0.57 | 0.11 | -6.69 | -1.53 | 2.59 | 30.3 | 31.3 | 29.3 |
| ARG447_ASP530 | 7.51 | -0.78 | -0.58 | -0.54 | -0.4 | -11.49 | -6.28 | 3.00 | 25.2 | 42.2 | 8.1 |
| LYS232_GLU218 | 5.6 | 0.73 | -0.54 | -3.67 | -0.03 | -7 | -4.91 | 3.16 | 26 | 44.2 | 7.8 |
| ARG172_ASP164 | 21.54 | -0.4 | 0.86 | -1.96 | -0.26 | -21.81 | -2.03 | 3.02 | 4.2 | 0 | 8.4 |
| LYS18_GLU21 | 2.54 | -1.03 | 0.03 | -0.07 | -0.05 | -2.72 | -1.3 | 3.39 | 60.6 | 55.4 | 65.8 |
| LYS108_ASP103 | 10.06 | 3.58 | -2.93 | -1.66 | -0.19 | -9.3 | -0.44 | 2.54 | 16.6 | 30.8 | 2.3 |
| LYS589_GLU94 | 10.63 | -0.6 | 0.11 | 0.37 | -0.27 | -15.05 | -4.81 | 2.64 | 20.2 | 10.5 | 29.9 |
| LYS465_ASP495 | 8.63 | 0.39 | -1.18 | -0.13 | -0.22 | -13.56 | -6.07 | 2.77 | 23.1 | 24.6 | 21.5 |
| LYS199_GLU104 | 6.54 | 2.71 | -1.41 | 0.23 | -0.15 | -10.23 | -2.31 | 2.96 | 33.6 | 17 | 50.3 |
| LYS204_ASP202 | 6.21 | 3.99 | -2.03 | -5.62 | -0.04 | -4.33 | -1.82 | 3.38 | 46.3 | 64.1 | 28.5 |
| ARG368_GLU366 | 3.95 | 0.05 | -0.25 | -0.09 | 0.09 | -3.56 | 0.19 | 2.91 | 56.6 | 68 | 45.2 |
| ARG182_GLU179 | 1.11 | -0.4 | 0.12 | 0.16 | 0.1 | -1.52 | -0.43 | 3.09 | 72.3 | 71.4 | 73.2 |
| ARG391_GLU393 | 3.19 | 0.17 | -0.81 | -0.03 | -0.01 | -6.98 | -4.47 | 2.95 | 57.5 | 73.8 | 41.3 |
| LYS315_ASP354 | 5.04 | 0.51 | -0.79 | -0.04 | 0.05 | -9.42 | -4.65 | 2.66 | 40 | 37.5 | 42.6 |
| ARG600_GLU603 | 13.53 | 4.63 | -6.41 | -8.27 | -0.15 | -12.86 | -9.53 | 2.50 | 16.5 | 32.5 | 0.5 |
| LYS388_ASP392 | 14.45 | -1.75 | -1.38 | 0.56 | 0.07 | -12.53 | -0.58 | 3.07 | 26.7 | 20 | 33.4 |
| LYS262_GLU273 | 2.12 | -1.19 | 0.23 | 0.15 | -0.13 | -6.38 | -5.2 | 2.71 | 38.9 | 29.7 | 48.1 |
| LYS85_GLU88 | 2.95 | -1.27 | 0.35 | -0.16 | -0.01 | -9.37 | -7.51 | 2.63 | 31.3 | 26.6 | 36 |
| LYS25_GLU29 | 1.95 | 0.12 | -0.3 | -0.05 | 0 | -5.61 | -3.89 | 3.23 | 60.2 | 62.5 | 58 |
| LYS255_GLU283 | 8.24 | -0.69 | 0.17 | -0.22 | -0.17 | -12.8 | -5.47 | 2.47 | 27.5 | 35.3 | 19.7 |

**Supplementary Table 10 | Component and net energy terms of network unit type of salt-bridge of 3ddu_A (707 residues) by network unit method (NUM).** Background energy is sub-divided into acidic (A), basic (B), and polar (P) parts. The net energy of the salt-bridge is the sum of the component (desolvation, bridge, and background) energy terms. Accessibility of partners of NU residues was extracted by NACCESS program whose average is the *ASA_av_*.

| Network unit (NU) type of  salt-bridge of 3ddu  (12 units) | Protein: 3ddu_A (707)  Energy terms (kcal/mol)  Method: Network unit method (NUM) | | | | | | | |
| --- | --- | --- | --- | --- | --- | --- | --- | --- |
|  | ASAav | *ΔΔG_dslv_* | *ΔΔG_brd_* | *ΔΔG_prt-A_* | *ΔΔG_prt-B_* | *ΔΔG_prt-P_* | *ΔΔG_prt-H_* | *ΔΔG_net_* |
| K40-D35-K196 | 45.97 | 7.38 | -9.88 | -8.4 | 3.39 | -0.68 | 0.33 | -8.19 |
| R128-D149-R643 | 22.67 | 14.8 | -19.01 | -4.7 | 6.47 | 0.5 | 0.11 | -1.93 |
| R85-E137-D159 | 36.4 | 8.75 | -11.14 | -0.43 | -1.4 | -0.13 | -0.05 | -4.35 |
| K390-E134-H180 | 35.87 | 2.3 | -9.73 | -5.25 | 2.42 | 0.72 | 0.18 | -9.55 |
| H213-D166-D222 | 1.43 | 25.59 | -23.32 | 2.23 | -1.61 | -4.58 | -0.5 | -1.7 |
| R505-D529-D446 | 16.83 | 22.32 | -26.76 | 6.69 | -5.84 | -11.01 | -0.87 | -14.6 |
| R567-D569-E535 | 36.8 | 8.69 | -15.92 | 2.01 | -2 | -0.68 | -1.06 | -7.89 |
| H515-E512-D598 | 3.37 | 26.81 | -24.59 | 9.22 | -16.19 | -2.76 | -0.35 | -7.52 |
| R252-D291-E289-K303 | 23.73 | 15.34 | -18 | -0.27 | 0.42 | 2.04 | 0.15 | -0.48 |
| K64-E691-R60-D695 | 28.7 | 12.91 | -26.7 | 4.13 | -5.88 | -0.94 | -0.28 | -16.49 |
| R306-E287-E323-H307 | 44.2 | 10.21 | -19.98 | -0.71 | 1.04 | 0.85 | 0.14 | -8.59 |
| K651-D26-K585-D582-E32 | 5.9 | 47.44 | -58.39 | 5.2 | -24.02 | -2.73 | -1.75 | -32.49 |

**Supplementary Table 11 | Component and net energy terms of network unit type of salt-bridge of 5t88_B (616 residues) by network unit method (NUM).** Background energy is sub-divided into acidic (A), basic (B), and polar (P) parts. The net energy of the salt-bridge is the sum of the component (desolvation, bridge, and background) energy terms. Accessibility of partners of NU residues was extracted by NACCESS program whose average is the *ASA_av_*.

| Network unit (NU) type of  salt-bridge of 5t88  (16 units) | *ASA_av_* Å^2^ | *ΔΔG_dslv_* | *ΔΔG_brd_* | *ΔΔG_prt-A_* | *ΔΔG_prt-B_* | *ΔΔG_prt-P_* | *ΔΔG_prt-H_* | *ΔΔG_net_* |
| --- | --- | --- | --- | --- | --- | --- | --- | --- |
| R26-E23-K576 | 31.03 | 13.47 | -18.53 | -0.57 | 0.69 | -0.17 | 0 | -5.11 |
| R602-E598-D36 | 56.2 | 4.63 | -8.3 | 3.17 | -2.81 | -1.16 | -0.1 | -4.47 |
| R124-E90-E136 | 32.97 | 10.93 | -18 | 1.59 | -1.2 | -1.45 | -0.04 | -8.13 |
| K291-D353-R289 | 37.97 | 7.19 | -7.7 | -6.17 | 2.85 | 0.24 | 0.35 | -3.6 |
| K313-E316-K303 | 37.67 | 4.44 | -9.31 | -5.33 | 3.11 | 0 | 0.3 | -7.1 |
| R347-E360-D320 | 29.57 | 14.79 | -22.61 | 1.21 | -2.2 | 0.23 | -0.24 | -8.58 |
| R422-E41-K421 | 40.6 | 10.16 | -19.69 | -1.66 | 1.79 | 0.26 | 0.04 | -9.14 |
| H442-E521-E439 | 10.3 | 19.71 | -21.59 | 6.11 | -16.41 | -4.66 | -0.64 | -16.84 |
| R432-D378-D456 | 16.27 | 22.23 | -27.8 | 6.87 | -7.16 | -11.92 | -0.32 | -17.78 |
| R490-D492-E462 | 46.4 | 6.76 | -16.2 | 1.35 | -2.85 | -0.17 | -0.66 | -11.11 |
| K463-D372-E467 | 37.03 | 5.86 | -12.24 | 2.26 | -3.87 | -0.07 | -0.06 | -8.06 |
| H510-E526-K511 | 38.4 | 5.57 | -7.97 | -7.15 | 9.5 | 0.50 | 1.5 | 0.45 |
| R585-D606-E587 | 13.23 | 13.36 | -20.36 | 3.08 | -4.61 | -5.42 | -0.48 | -13.95 |
| R158-D119-K159-E120-K138 | 50.4 | 6.93 | -10.52 | -2.95 | 2.72 | 0.45 | 0.35 | -3.37 |
| R334-E350-K352-E333 | 30.4 | 11.25 | -16.74 | -3.11 | -0.16 | 0.19 | 0.1 | -8.56 |
| R508-E9-D3-K570-D505 | 8.14 | 38.93 | -49.93 | 3.77 | -16.31 | -6.85 | -2.19 | -30.38 |

**Supplementary Table 12 | Residue specific details of the microenvironment for isolated (28 pairs) and network unit type of salt-bridges (16 units) of 5t88_B.** Microenvironment (ME) residue can participate in isolated (IP) or network (NU) or both(IPNU) types of salt-bridge (SB). Thus, types of ME are three types: IP_ME (ME for IP type of SB), NU_ME (ME for NU type of SB), and IPNU_ME ME for both IP and NU types of SB). Each residue (row-wise) is presented with residue name, residue ID as per the PDB file (5t88_B). ME_Energy is the interaction energy between the ME residue with the positive and negative partners of the concerned salt-bridge. An ME that can be either an IP partner (IP_ME) or an NU partner (NU_ME) or a non-salt-bridge ME partner (nSBME) by itself can participate in the IP or NU or both types salt-bridge's microenvironment as a ME candidate. Residue-specific total interaction energy (ME_Energy) with the partners of salt-bridge is expressed in kJ/mol. Residue side-chain accessibility (ASA), type of secondary structure (Coil C, Helix H, and strand S) are also shown. Again, an ME residue can be used as an ME candidate for multiple salt-bridges. It has been denoted by the Times Used parameter. Only those residues were considered as ME-residue whose interaction energy was either greater than 0.75 kJ/mol (unstable) or less than -0.75 kJ/mol (stable). If a residue is ME for multiple times, the sum of the energy was not used as screening criteria.

| **Type of ME** | **Residue name** | **Residue ID** | **ME-Energy**  **(kJ/mol)** | **ASA Å^2^** | **SECONDARY**  **STRUCTURE**  **TYPE** | **Times Used** | **Type of partner**  **SB (IP or NU)**  **nSB (non SB)** |
| --- | --- | --- | --- | --- | --- | --- | --- |
| NU_ME | GLU | 9 | -6.31 | 10.4 | H | 1 | NU |
| NU_ME | ASP | 13 | 0.84 | 16.3 | H | 1 | IP |
| NU_ME | ASP | 36 | 4.18 | 71.2 | H | 1 | NU |
| NU_ME | LYS | 37 | 1.19 | 60.7 | H | 1 | IP |
| NU_ME | GLU | 136 | -1.29 | 41.1 | S | 1 | NU |
| NU_ME | LYS | 138 | -1.06 | 55.2 | C | 1 | NU |
| NU_ME | GLU | 305 | -10.29 | 3.4 | S | 2 | IP |
| NU_ME | LYS | 315 | 3.15 | 37.5 | S | 1 | IP |
| NU_ME | GLU | 333 | -1.77 | 60 | C | 1 | NU |
| NU_ME | LYS | 352 | 1.13 | 30.2 | C | 1 | NU |
| NU_ME | ASP | 354 | -2.95 | 42.6 | C | 1 | IP |
| NU_ME | GLU | 371 | -0.39 | 31.8 | S | 2 | IP |
| NU_ME | HIS | 383 | -5.04 | 0 | S | 3 | IP |
| NU_ME | LYS | 388 | -3.93 | 20 | S | 1 | IP |
| NU_ME | ARG | 432 | -1.33 | 7.2 | C | 1 | NU |
| NU_ME | ARG | 447 | -2.36 | 42.2 | H | 1 | IP |
| NU_ME | ASP | 456 | 2.31 | 0 | H | 1 | NU |
| NU_ME | GLU | 462 | 1.26 | 46.9 | H | 1 | NU |
| NU_ME | LYS | 465 | -1.99 | 24.6 | H | 1 | IP |
| NU_ME | ASP | 495 | 1.53 | 21.5 | C | 1 | IP |
| NU_ME | ARG | 508 | 8.08 | 22.8 | H | 1 | NU |
| NU_ME | LYS | 511 | 0.91 | 44.3 | H | 1 | NU |
| NU_ME | GLU | 526 | 1.26 | 61.1 | C | 1 | NU |
| NU_ME | ASP | 530 | 1.13 | 8.1 | H | 3 | IP |
| NU_ME | LYS | 547 | -1.32 | 31.3 | C | 1 | IP |
| NU_ME | LYS | 574 | -4.11 | 27.8 | H | 1 | IP |
| NU_ME | GLU | 577 | 2.70 | 37.5 | H | 1 | IP |
| NU_ME | GLU | 598 | 3.27 | 53.2 | H | 1 | NU |
| IP_ME | GLU | 23 | -0.76 | 7.5 | H | 1 | NU |
| IP_ME | ARG | 55 | -3.33 | 28.1 | S | 2 | IP |
| IP_ME | ARG | 70 | 1.70 | 29.8 | S | 1 | IP |
| IP_ME | ASP | 83 | -1.37 | 8.2 | S | 1 | IP |
| IP_ME | GLU | 90 | 0.93 | 46.6 | H | 1 | NU |
| IP_ME | ASP | 103 | 3.58 | 2.3 | C | 1 | IP |
| IP_ME | GLU | 104 | 6.96 | 50.3 | C | 3 | IP |
| IP_ME | LYS | 108 | -1.22 | 30.8 | S | 1 | IP |
| IP_ME | ASP | 164 | -1.10 | 8.4 | C | 1 | IP |
| IP_ME | ARG | 172 | 1.79 | 0 | S | 1 | IP |
| IP_ME | LYS | 199 | -3.62 | 17 | S | 2 | IP |
| IP_ME | LYS | 303 | -2.43 | 30.5 | S | 1 | NU |
| IP_ME | LYS | 313 | -4.82 | 47.9 | S | 1 | NU |
| IP_ME | GLU | 316 | 4.33 | 34.6 | S | 1 | NU |
| IP_ME | GLU | 439 | -2.42 | 28.7 | H | 1 | NU |
| IP_ME | GLU | 467 | -1.84 | 42.7 | H | 1 | NU |
| IP_ME | ASP | 492 | -1.56 | 56.1 | H | 1 | NU |
| IPNU_ME | ASP | 3 | -4.65 | 5.4 | C | 2 | NU |
| IPNU_ME | LYS | 85 | -3.06 | 26.6 | H | 3 | IP |
| IPNU_ME | GLU | 88 | 3.88 | 36 | H | 3 | IP |
| IPNU_ME | GLU | 94 | 3.38 | 29.9 | C | 3 | IP |
| IPNU_ME | ARG | 124 | -0.05 | 11.2 | S | 2 | NU |
| IPNU_ME | ARG | 155 | 2.14 | 22.7 | S | 2 | IP |
| IPNU_ME | LYS | 255 | -0.30 | 35.3 | H | 2 | IP |
| IPNU_ME | GLU | 283 | -0.20 | 19.7 | S | 2 | IP |
| IPNU_ME | ARG | 296 | 7.57 | 4.4 | S | 2 | IP |
| IPNU_ME | ASP | 329 | 2.82 | 21.6 | S | 3 | IP |
| IPNU_ME | ARG | 334 | -0.36 | 21.7 | C | 2 | NU |
| IPNU_ME | GLU | 350 | -0.41 | 9.7 | S | 2 | NU |
| IPNU_ME | ASP | 372 | -1.61 | 44.2 | S | 3 | NU |
| IPNU_ME | ASP | 392 | 3.28 | 33.4 | C | 2 | IP |
| IPNU_ME | GLU | 393 | 0.35 | 41.3 | C | 2 | IP |
| IPNU_ME | HIS | 442 | -1.02 | 2.1 | H | 2 | IP |
| IPNU_ME | LYS | 463 | 0.59 | 24.2 | H | 4 | NU |
| IPNU_ME | ARG | 490 | -0.17 | 36.2 | H | 2 | NU |
| IPNU_ME | ASP | 505 | -9.99 | 1.8 | C | 3 | NU |
| IPNU_ME | HIS | 510 | -9.21 | 9.8 | H | 3 | NU |
| IPNU_ME | GLU | 521 | 1.94 | 0.1 | H | 4 | NU |
| IPNU_ME | LYS | 570 | 7.15 | 0.3 | H | 2 | NU |
| IPNU_ME | ARG | 585 | -19.70 | 13 | S | 2 | NU |
| IPNU_ME | GLU | 587 | 9.84 | 13.4 | S | 3 | NU |
| IPNU_ME | LYS | 589 | -5.85 | 10.5 | C | 3 | IP |
| IPNU_ME | ARG | 600 | -0.06 | 32.5 | H | 2 | IP |
| IPNU_ME | ARG | 602 | -5.11 | 44.2 | H | 2 | NU |
| IPNU_ME | GLU | 603 | -2.74 | 0.5 | H | 3 | IP |
| IPNU_ME | ASP | 606 | 7.82 | 13.3 | H | 2 | NU |
| NU_ME | MET | 1 | 1.65 | 56.1 | C | 1 | nSBME |
| NU_ME | GLU | 2 | 1.17 | 102 | C | 1 | nSBME |
| NU_ME | PRO | 4 | -4.70 | 61.8 | C | 1 | nSBME |
| NU_ME | TRP | 7 | 0.77 | 27.9 | H | 1 | nSBME |
| NU_ME | ARG | 28 | -5.36 | 67.6 | H | 2 | nSBME |
| NU_ME | SER | 35 | -19.47 | 6.7 | H | 1 | nSBME |
| NU_ME | TYR | 111 | -5.59 | 6 | S | 1 | nSBME |
| NU_ME | GLU | 160 | -0.91 | 83 | C | 1 | nSBME |
| NU_ME | TRP | 214 | -2.03 | 44 | C | 1 | nSBME |
| NU_ME | PRO | 239 | -0.75 | 40.5 | C | 1 | nSBME |
| NU_ME | GLU | 241 | 1.72 | 68.2 | S | 1 | nSBME |
| NU_ME | GLU | 279 | -5.66 | 58.6 | C | 1 | nSBME |
| NU_ME | ASP | 290 | -8.30 | 71 | C | 1 | nSBME |
| NU_ME | TYR | 339 | -1.49 | 0.1 | S | 1 | nSBME |
| NU_ME | TYR | 349 | 1.45 | 9.3 | S | 1 | nSBME |
| NU_ME | LYS | 362 | -5.37 | 50.1 | C | 1 | nSBME |
| NU_ME | THR | 375 | -0.81 | 51.9 | S | 1 | nSBME |
| NU_ME | SER | 376 | -18.15 | 0 | C | 1 | nSBME |
| NU_ME | LYS | 377 | -1.55 | 72.9 | C | 1 | nSBME |
| NU_ME | GLY | 379 | -0.82 | 0 | C | 1 | nSBME |
| NU_ME | THR | 380 | -20.55 | 19.2 | C | 1 | nSBME |
| NU_ME | GLY | 400 | -0.80 | 0 | C | 1 | nSBME |
| NU_ME | GLY | 402 | -2.24 | 0 | C | 2 | nSBME |
| NU_ME | GLY | 403 | -3.86 | 0 | C | 1 | nSBME |
| NU_ME | PHE | 404 | 2.85 | 16.4 | C | 1 | nSBME |
| NU_ME | ASN | 405 | -3.86 | 40.4 | C | 1 | nSBME |
| NU_ME | GLY | 433 | -2.63 | 0 | C | 2 | nSBME |
| NU_ME | GLY | 445 | -2.11 | 0 | H | 2 | nSBME |
| NU_ME | MET | 446 | -1.07 | 8.2 | H | 1 | nSBME |
| NU_ME | LYS | 450 | -32.94 | 0.6 | H | 4 | nSBME |
| NU_ME | ASN | 452 | -0.91 | 31 | H | 1 | nSBME |
| NU_ME | ASP | 455 | 6.55 | 16.6 | H | 1 | nSBME |
| NU_ME | LYS | 466 | -3.23 | 68.9 | H | 2 | nSBME |
| NU_ME | ASN | 478 | 1.98 | 1.6 | H | 1 | nSBME |
| NU_ME | MET | 506 | 0.86 | 0 | C | 1 | nSBME |
| NU_ME | PHE | 509 | -2.34 | 0 | H | 1 | nSBME |
| NU_ME | GLY | 515 | 1.28 | 0 | H | 1 | nSBME |
| NU_ME | SER | 516 | 1.56 | 57.1 | H | 1 | nSBME |
| NU_ME | PRO | 520 | -0.75 | 49.8 | H | 1 | nSBME |
| NU_ME | TYR | 522 | -1.00 | 0.4 | H | 1 | nSBME |
| NU_ME | ASN | 524 | -2.02 | 45.4 | C | 1 | nSBME |
| NU_ME | SER | 538 | 3.30 | 0 | H | 1 | nSBME |
| NU_ME | PRO | 539 | -2.08 | 0 | H | 1 | nSBME |
| NU_ME | TYR | 540 | -29.60 | 5 | H | 1 | nSBME |
| NU_ME | ASN | 542 | -1.20 | 31.6 | H | 1 | nSBME |
| NU_ME | LYS | 548 | -0.83 | 93.3 | C | 1 | nSBME |
| NU_ME | ASP | 561 | -1.52 | 70.1 | C | 1 | nSBME |
| NU_ME | ARG | 562 | -0.46 | 64.1 | C | 2 | nSBME |
| NU_ME | HIS | 564 | -2.52 | 6.4 | C | 2 | nSBME |
| NU_ME | HIS | 567 | -9.69 | 0.8 | H | 3 | nSBME |
| NU_ME | TYR | 583 | -1.44 | 12.9 | S | 1 | nSBME |
| NU_ME | THR | 588 | -1.22 | 54.2 | C | 1 | nSBME |
| NU_ME | THR | 605 | -1.37 | 0 | H | 1 | nSBME |
| IP_ME | TYR | 5 | -20.00 | 16.1 | H | 1 | nSBME |
| IP_ME | GLU | 14 | 0.92 | 96 | H | 1 | nSBME |
| IP_ME | GLU | 22 | -5.47 | 62.4 | H | 1 | nSBME |
| IP_ME | LYS | 58 | -0.97 | 67 | C | 1 | nSBME |
| IP_ME | LYS | 59 | -6.13 | 42.7 | C | 1 | nSBME |
| IP_ME | LYS | 68 | 1.88 | 66.9 | C | 1 | nSBME |
| IP_ME | ASP | 69 | -21.82 | 42.8 | C | 2 | nSBME |
| IP_ME | SER | 84 | 3.60 | 11.1 | H | 1 | nSBME |
| IP_ME | ARG | 89 | -1.71 | 66.4 | H | 1 | nSBME |
| IP_ME | ASP | 93 | 1.59 | 49.1 | C | 1 | nSBME |
| IP_ME | PHE | 100 | -1.38 | 0.6 | S | 1 | nSBME |
| IP_ME | THR | 102 | -3.91 | 2.4 | S | 1 | nSBME |
| IP_ME | GLU | 105 | 10.16 | 49.1 | C | 2 | nSBME |
| IP_ME | GLY | 106 | -2.51 | 0 | C | 1 | nSBME |
| IP_ME | GLU | 107 | 2.26 | 69 | C | 1 | nSBME |
| IP_ME | GLU | 135 | -1.14 | 28.7 | S | 1 | nSBME |
| IP_ME | THR | 145 | 0.77 | 10 | S | 1 | nSBME |
| IP_ME | PHE | 153 | -0.81 | 1.7 | S | 1 | nSBME |
| IP_ME | TYR | 157 | 5.75 | 14.7 | S | 2 | nSBME |
| IP_ME | THR | 162 | -14.20 | 0 | C | 1 | nSBME |
| IP_ME | PRO | 163 | -1.03 | 39.3 | C | 1 | nSBME |
| IP_ME | PHE | 174 | -1.71 | 3.5 | S | 1 | nSBME |
| IP_ME | LYS | 176 | -1.41 | 28.8 | S | 1 | nSBME |
| IP_ME | GLY | 186 | 2.10 | 0 | C | 1 | nSBME |
| IP_ME | GLU | 187 | 1.27 | 81.7 | C | 1 | nSBME |
| IP_ME | TYR | 193 | -0.76 | 18.6 | S | 1 | nSBME |
| IP_ME | ARG | 198 | -3.87 | 46 | S | 3 | nSBME |
| IP_ME | SER | 200 | -20.24 | 0 | C | 1 | nSBME |
| IP_ME | SER | 201 | -1.69 | 26.5 | C | 1 | nSBME |
| IP_ME | GLY | 203 | -0.88 | 0 | C | 1 | nSBME |
| IP_ME | GLN | 216 | -9.66 | 36.3 | S | 1 | nSBME |
| IP_ME | GLY | 217 | -0.80 | 0 | S | 1 | nSBME |
| IP_ME | TYR | 220 | 1.06 | 24.8 | S | 1 | nSBME |
| IP_ME | ASP | 225 | -0.76 | 68.2 | C | 1 | nSBME |
| IP_ME | SER | 235 | -3.97 | 61.5 | S | 1 | nSBME |
| IP_ME | GLY | 261 | 2.97 | 0 | C | 2 | nSBME |
| IP_ME | PRO | 276 | -0.81 | 69 | C | 1 | nSBME |
| IP_ME | GLU | 277 | -6.11 | 38.9 | C | 3 | nSBME |
| IP_ME | GLY | 278 | 1.17 | 0 | C | 1 | nSBME |
| IP_ME | PRO | 281 | 1.17 | 0.3 | C | 1 | nSBME |
| IP_ME | SER | 301 | 1.10 | 19.2 | S | 1 | nSBME |
| IP_ME | TYR | 302 | -1.25 | 4.5 | S | 1 | nSBME |
| IP_ME | PRO | 322 | -1.22 | 23.9 | C | 1 | nSBME |
| IP_ME | PHE | 373 | -2.61 | 36.7 | S | 1 | nSBME |
| IP_ME | PHE | 385 | -4.88 | 0 | S | 1 | nSBME |
| IP_ME | PRO | 410 | 1.02 | 0 | C | 1 | nSBME |
| IP_ME | THR | 425 | -1.44 | 0.7 | S | 1 | nSBME |
| IP_ME | ASN | 430 | -11.77 | 0 | C | 1 | nSBME |
| IP_ME | SER | 435 | -1.45 | 12.7 | C | 1 | nSBME |
| IP_ME | TYR | 469 | 4.34 | 7.2 | C | 1 | nSBME |
| IP_ME | LYS | 470 | -0.97 | 49.9 | S | 1 | nSBME |
| IP_ME | GLY | 500 | -0.79 | 0 | S | 1 | nSBME |
| IP_ME | TYR | 501 | -28.23 | 6.3 | C | 1 | nSBME |
| IP_ME | GLY | 523 | -2.20 | 0 | H | 1 | nSBME |
| IP_ME | ASP | 527 | 3.04 | 43.1 | H | 1 | nSBME |
| IP_ME | LYS | 536 | 0.79 | 81.1 | H | 1 | nSBME |
| IP_ME | PRO | 545 | -1.92 | 69.8 | C | 1 | nSBME |
| IP_ME | LYS | 546 | -0.88 | 83.3 | C | 1 | nSBME |
| IP_ME | TYR | 549 | -2.72 | 1.9 | C | 1 | nSBME |
| IP_ME | TYR | 555 | -2.23 | 1 | S | 1 | nSBME |
| IP_ME | GLY | 557 | -1.17 | 0 | S | 1 | nSBME |
| IP_ME | MET | 593 | -1.03 | 28.4 | H | 1 | nSBME |
| IP_ME | SER | 596 | 2.81 | 25.6 | H | 1 | nSBME |
| IPNU_ME | GLU | 86 | 4.21 | 66 | H | 2 | nSBME |
| IPNU_ME | ASP | 244 | 9.51 | 6.3 | S | 3 | nSBME |
| IPNU_ME | GLU | 256 | -0.43 | 26.9 | H | 3 | nSBME |
| IPNU_ME | LYS | 258 | -8.11 | 38.7 | H | 2 | nSBME |
| IPNU_ME | HIS | 299 | 7.45 | 38.7 | C | 3 | nSBME |
| IPNU_ME | TYR | 307 | -21.87 | 6.4 | S | 2 | nSBME |
| IPNU_ME | LYS | 330 | -3.48 | 23.5 | S | 4 | nSBME |
| IPNU_ME | ASP | 331 | -9.04 | 39.5 | C | 3 | nSBME |
| IPNU_ME | GLU | 332 | 0.55 | 71.8 | C | 2 | nSBME |
| IPNU_ME | ARG | 356 | -4.75 | 59.5 | S | 2 | nSBME |
| IPNU_ME | GLU | 370 | -2.81 | 50.9 | S | 3 | nSBME |
| IPNU_ME | LYS | 381 | -2.16 | 54.9 | S | 3 | nSBME |
| IPNU_ME | LYS | 394 | -10.33 | 43.2 | C | 2 | nSBME |
| IPNU_ME | ARG | 395 | -3.18 | 35.4 | S | 4 | nSBME |
| IPNU_ME | GLU | 436 | 1.24 | 2 | C | 4 | nSBME |
| IPNU_ME | ARG | 443 | -7.25 | 60.3 | H | 2 | nSBME |
| IPNU_ME | GLU | 448 | -5.02 | 60.9 | H | 2 | nSBME |
| IPNU_ME | ARG | 476 | -3.85 | 40.5 | S | 2 | nSBME |
| IPNU_ME | PRO | 491 | 0.29 | 5.9 | H | 2 | nSBME |
| IPNU_ME | PRO | 502 | -2.05 | 1.2 | C | 2 | nSBME |
| IPNU_ME | PRO | 525 | 2.88 | 0.9 | C | 3 | nSBME |
| IPNU_ME | ARG | 531 | -1.84 | 26.4 | H | 3 | nSBME |
| IPNU_ME | HIS | 541 | -18.03 | 30.3 | H | 3 | nSBME |
| IPNU_ME | HIS | 559 | -1.68 | 45.7 | C | 3 | nSBME |
| IPNU_ME | ASP | 560 | 2.35 | 18.9 | C | 5 | nSBME |
| IPNU_ME | HIS | 592 | -2.01 | 33.8 | H | 2 | nSBME |
| IPNU_ME | THR | 599 | -3.56 | 18.7 | H | 2 | nSBME |

**Supplementary Table 13 | Residue specific details of the microenvironment for isolated (21 pairs) and network unit type of salt-bridges (12 units) of 3ddu_A.** Microenvironment (ME) residue can participate in isolated (IP) or network (NU) or both(IPNU) types of salt-bridge (SB). Thus, types of ME are three types: IP_ME (ME for IP type of SB), NU_ME (ME for NU type of SB), and IPNU_ME ME for both IP and NU types of SB). Each residue (row-wise) is presented with residue name, residue ID as per the PDB file (3ddu_A). ME_Energy is the interaction energy between the ME residue with the positive and negative partners of the concerned salt-bridge. An ME that can be either an IP partner (IP_ME) or NU partner (NU_ME) or a non-salt-bridge ME partner (nSBME) by itself can participate in the IP or NU or both types salt-bridge's microenvironment as a ME candidate. Residue-specific total interaction energy (ME_Energy) with the partners of salt-bridge is expressed in kJ/mol. Residue side-chain accessibility (ASA), type of secondary structure (Coil C, Helix H, and strand S) are also shown. Again, an ME residue can be used as an ME candidate for multiple salt-bridges. It has been denoted by the Times Used parameter. Only those residues were considered as ME-residue whose interaction energy was either greater than 0.75 kJ/mol (unstable) or less than -0.75 kJ/mol (stable). If a residue is ME for multiple times, the sum of the energy was not used as screening criteria.

| **Type of ME** | **Residue name** | **Residue ID** | **ME-Energy**  **(kJ/mol)** | **ASA Å^2^** | **Secondary**  **structure**  **type** | **Times Used** | **Type of partner**  **i) SB (IP or NU)**  **ii) nSB (non SB)** |
| --- | --- | --- | --- | --- | --- | --- | --- |
| NU_ME | ARG | 11 | -2.84 | 22.4 | C | 1 | IP |
| NU_ME | ARG | 111 | 3.18 | 54.3 | S | 1 | IP |
| NU_ME | ARG | 252 | 0.09 | 38 | S | 2 | NU |
| NU_ME | ARG | 260 | -0.80 | 23.1 | S | 1 | IP |
| NU_ME | ARG | 306 | -1.08 | 21 | C | 1 | NU |
| NU_ME | ARG | 567 | -1.01 | 37.6 | H | 1 | NU |
| NU_ME | ASP | 104 | -6.40 | 61.4 | C | 1 | IP |
| NU_ME | ASP | 265 | -0.86 | 13 | S | 1 | IP |
| NU_ME | ASP | 291 | -0.02 | 33.2 | S | 2 | NU |
| NU_ME | ASP | 528 | 7.68 | 17.5 | H | 2 | IP |
| NU_ME | ASP | 598 | 2.61 | 0.4 | H | 3 | NU |
| NU_ME | ASP | 603 | 1.22 | 70 | C | 1 | IP |
| NU_ME | GLU | 13 | 1.51 | 60.7 | C | 1 | IP |
| NU_ME | GLU | 289 | 1.39 | 11.3 | C | 2 | NU |
| NU_ME | GLU | 296 | -1.16 | 60.1 | S | 1 | IP |
| NU_ME | GLU | 44 | -1.13 | 66.1 | H | 1 | IP |
| NU_ME | GLU | 512 | 0.63 | 8.7 | H | 3 | NU |
| NU_ME | HIS | 307 | -0.83 | 64.4 | C | 1 | NU |
| NU_ME | HIS | 515 | -1.07 | 1 | H | 2 | NU |
| NU_ME | HIS | 587 | -9.01 | 16.2 | H | 2 | IP |
| NU_ME | LYS | 303 | -0.65 | 12.4 | S | 2 | NU |
| NU_ME | LYS | 389 | 2.20 | 19.6 | C | 1 | IP |
| NU_ME | LYS | 445 | -2.50 | 73.2 | C | 2 | IP |
| IP_ME | ARG | 420 | 1.60 | 75.8 | S | 1 | IP |
| IP_ME | ARG | 60 | -0.93 | 14.5 | H | 1 | NU |
| IP_ME | ARG | 85 | -3.34 | 9.9 | S | 1 | NU |
| IP_ME | ASP | 122 | -2.52 | 37 | C | 1 | IP |
| IP_ME | ASP | 26 | -3.49 | 3.6 | C | 2 | NU |
| IP_ME | ASP | 336 | 1.93 | 31.5 | C | 1 | IP |
| IP_ME | ASP | 356 | -2.00 | 20.1 | C | 1 | IP |
| IP_ME | ASP | 446 | 2.36 | 41.3 | C | 1 | NU |
| IP_ME | ASP | 695 | 2.42 | 15.2 | H | 1 | NU |
| IP_ME | GLU | 32 | 1.03 | 19.6 | H | 1 | NU |
| IP_ME | GLU | 418 | -0.87 | 44.9 | C | 1 | IP |
| IP_ME | HIS | 180 | 1.01 | 21.4 | C | 1 | NU |
| IP_ME | HIS | 355 | -4.31 | 49 | S | 1 | IP |
| IP_ME | HIS | 409 | 0.90 | 21.3 | S | 1 | IP |
| IP_ME | LYS | 390 | 1.21 | 56.4 | C | 1 | NU |
| IP_ME | LYS | 585 | 1.84 | 5.9 | H | 1 | NU |
| IP_ME | LYS | 651 | 1.28 | 0.1 | H | 1 | NU |
| IPNU_ME | ARG | 128 | -6.79 | 25.2 | S | 2 | NU |
| IPNU_ME | ARG | 505 | -4.61 | 9.2 | C | 2 | NU |
| IPNU_ME | ARG | 643 | -5.41 | 38.2 | C | 3 | NU |
| IPNU_ME | ASP | 149 | 14.32 | 4.6 | C | 4 | NU |
| IPNU_ME | ASP | 529 | 9.72 | 0 | H | 3 | NU |
| IPNU_ME | ASP | 582 | -4.44 | 0.3 | C | 3 | NU |
| IPNU_ME | ASP | 641 | 14.90 | 0 | C | 8 | IP |
| IPNU_ME | ASP | 642 | 21.11 | 1.4 | C | 5 | IP |
| IPNU_ME | ASP | 675 | 1.34 | 30.2 | S | 7 | IP |
| IPNU_ME | GLU | 535 | 2.70 | 42.3 | H | 2 | NU |
| IPNU_ME | GLU | 692 | 6.88 | 21.7 | H | 4 | IP |
| IPNU_ME | HIS | 680 | -4.48 | 22.6 | C | 5 | IP |
| IPNU_ME | LYS | 172 | -13.38 | 0.2 | C | 5 | IP |
| IPNU_ME | LYS | 677 | 2.32 | 49.8 | C | 2 | IP |
| IPNU_ME | LYS | 684 | -10.39 | 14.6 | C | 5 | IP |
| IPNU_ME | LYS | 688 | -0.23 | 21.2 | H | 6 | IP |
| IPNU_ME | ARG | 488 | -1.65 | 25.3 | H | 3 | nSBME |
| IPNU_ME | ASN | 205 | -18.71 | 0.7 | C | 2 | nSBME |
| IPNU_ME | ASP | 12 | 2.57 | 35.8 | C | 2 | nSBME |
| IPNU_ME | ASP | 198 | -8.83 | 45.5 | C | 3 | nSBME |
| IPNU_ME | ASP | 242 | -17.42 | 28.8 | C | 3 | nSBME |
| IPNU_ME | ASP | 243 | -7.94 | 39.6 | C | 3 | nSBME |
| IPNU_ME | ASP | 391 | 2.40 | 12.6 | C | 2 | nSBME |
| IPNU_ME | ASP | 639 | 1.29 | 36.3 | C | 7 | nSBME |
| IPNU_ME | GLN | 531 | -3.08 | 11.7 | H | 3 | nSBME |
| IPNU_ME | GLU | 201 | -6.35 | 6 | C | 5 | nSBME |
| IPNU_ME | GLU | 253 | -1.12 | 34.7 | C | 2 | nSBME |
| IPNU_ME | GLU | 37 | -2.17 | 90.2 | H | 2 | nSBME |
| IPNU_ME | GLU | 509 | 11.02 | 1.1 | C | 4 | nSBME |
| IPNU_ME | GLY | 147 | 0.05 | 0 | C | 2 | nSBME |
| IPNU_ME | HIS | 20 | -3.32 | 24.2 | C | 2 | nSBME |
| IPNU_ME | HIS | 22 | -3.44 | 47.6 | S | 2 | nSBME |
| IPNU_ME | HIS | 593 | 5.04 | 9 | H | 4 | nSBME |
| IPNU_ME | HIS | 607 | -1.85 | 28.1 | H | 3 | nSBME |
| IPNU_ME | HIS | 618 | -39.57 | 8.8 | H | 4 | nSBME |
| IPNU_ME | HIS | 640 | -5.50 | 17.6 | C | 5 | nSBME |
| IPNU_ME | HIS | 648 | -29.85 | 1.1 | H | 6 | nSBME |
| IPNU_ME | HIS | 673 | -11.95 | 23.7 | S | 5 | nSBME |
| IPNU_ME | LYS | 523 | -36.93 | 1.3 | H | 5 | nSBME |
| IPNU_ME | LYS | 588 | -1.62 | 47.9 | H | 2 | nSBME |
| IPNU_ME | LYS | 81 | -3.41 | 38.1 | S | 2 | nSBME |
| IPNU_ME | LYS | 82 | 0.15 | 21.2 | S | 2 | nSBME |
| IPNU_ME | PRO | 646 | -3.65 | 18.1 | H | 3 | nSBME |
| IPNU_ME | SER | 602 | -1.08 | 0.8 | C | 2 | nSBME |
| IPNU_ME | THR | 590 | 0.10 | 1 | C | 3 | nSBME |
| IPNU_ME | TYR | 19 | -0.64 | 13.2 | S | 2 | nSBME |
| IP_ME | ARG | 170 | 4.15 | 22.4 | S | 1 | nSBME |
| IP_ME | ARG | 493 | 2.35 | 51.3 | H | 1 | nSBME |
| IP_ME | ARG | 547 | 1.29 | 41.6 | C | 1 | nSBME |
| IP_ME | ARG | 662 | 0.99 | 62.5 | C | 1 | nSBME |
| IP_ME | ASN | 295 | -2.37 | 0.2 | S | 1 | nSBME |
| IP_ME | ASP | 317 | 1.31 | 34.9 | S | 1 | nSBME |
| IP_ME | CYS | 601 | 1.77 | 22.6 | C | 1 | nSBME |
| IP_ME | GLN | 193 | 0.88 | 17.8 | C | 1 | nSBME |
| IP_ME | GLN | 208 | 1.06 | 1.3 | C | 1 | nSBME |
| IP_ME | GLN | 268 | -1.94 | 68.7 | H | 1 | nSBME |
| IP_ME | GLN | 524 | -17.13 | 43.1 | H | 1 | nSBME |
| IP_ME | GLN | 566 | 2.02 | 22.3 | H | 1 | nSBME |
| IP_ME | GLN | 577 | -11.76 | 22 | S | 1 | nSBME |
| IP_ME | GLN | 97 | -0.90 | 12 | C | 1 | nSBME |
| IP_ME | GLU | 231 | -1.05 | 52.4 | C | 1 | nSBME |
| IP_ME | GLU | 269 | 2.23 | 8.1 | H | 1 | nSBME |
| IP_ME | GLU | 416 | 1.97 | 83.1 | C | 1 | nSBME |
| IP_ME | GLU | 65 | 1.45 | 64 | H | 1 | nSBME |
| IP_ME | GLY | 123 | -0.90 | 0 | C | 1 | nSBME |
| IP_ME | GLY | 199 | -2.54 | 0 | C | 1 | nSBME |
| IP_ME | GLY | 592 | 1.07 | 0 | H | 1 | nSBME |
| IP_ME | GLY | 679 | -3.43 | 0 | C | 2 | nSBME |
| IP_ME | HIS | 333 | -3.26 | 55.3 | C | 1 | nSBME |
| IP_ME | HIS | 364 | -0.79 | 7.3 | S | 1 | nSBME |
| IP_ME | HIS | 456 | 4.41 | 6.9 | S | 1 | nSBME |
| IP_ME | HIS | 494 | 12.91 | 6.9 | H | 1 | nSBME |
| IP_ME | LYS | 281 | -5.43 | 50.6 | S | 1 | nSBME |
| IP_ME | LYS | 358 | -0.62 | 29.5 | S | 2 | nSBME |
| IP_ME | LYS | 373 | -0.83 | 28.5 | S | 1 | nSBME |
| IP_ME | LYS | 457 | 0.98 | 67.4 | S | 1 | nSBME |
| IP_ME | LYS | 461 | -1.31 | 84.6 | C | 1 | nSBME |
| IP_ME | PHE | 286 | -3.14 | 12.3 | C | 1 | nSBME |
| IP_ME | PHE | 395 | -4.19 | 1.5 | S | 1 | nSBME |
| IP_ME | PHE | 586 | -2.56 | 0 | H | 1 | nSBME |
| IP_ME | PRO | 467 | 1.61 | 16 | C | 1 | nSBME |
| IP_ME | PRO | 685 | 1.58 | 2.8 | H | 3 | nSBME |
| IP_ME | PRO | 710 | -1.37 | 116.7 | C | 1 | nSBME |
| IP_ME | SER | 120 | 4.80 | 9.4 | H | 1 | nSBME |
| IP_ME | SER | 148 | -11.19 | 2.5 | C | 2 | nSBME |
| IP_ME | SER | 465 | -7.00 | 52.7 | C | 1 | nSBME |
| IP_ME | SER | 554 | -4.11 | 22.9 | H | 1 | nSBME |
| IP_ME | SER | 649 | -1.47 | 1.8 | H | 1 | nSBME |
| IP_ME | THR | 124 | -15.82 | 3.1 | C | 1 | nSBME |
| IP_ME | THR | 294 | -11.50 | 0.5 | S | 1 | nSBME |
| IP_ME | THR | 543 | 1.29 | 0 | C | 1 | nSBME |
| IP_ME | THR | 596 | 1.19 | 21.6 | H | 1 | nSBME |
| IP_ME | THR | 637 | 3.23 | 1.1 | S | 1 | nSBME |
| IP_ME | THR | 676 | 0.97 | 74.6 | C | 1 | nSBME |
| IP_ME | TRP | 150 | -4.37 | 8.4 | C | 1 | nSBME |
| IP_ME | TRP | 234 | -1.17 | 14.3 | C | 1 | nSBME |
| IP_ME | TRP | 262 | -6.61 | 9.7 | S | 1 | nSBME |
| IP_ME | TYR | 246 | 2.02 | 5.3 | S | 1 | nSBME |
| IP_ME | TYR | 28 | -6.38 | 1.8 | H | 1 | nSBME |
| IP_ME | TYR | 510 | -9.81 | 14.4 | H | 1 | nSBME |
| IP_ME | TYR | 658 | -1.89 | 36.8 | H | 1 | nSBME |
| IP_ME | TYR | 86 | -17.98 | 13.1 | S | 1 | nSBME |
| NU_ME | ARG | 312 | 2.23 | 27.6 | S | 1 | nSBME |
| NU_ME | ARG | 98 | 2.23 | 54.2 | C | 1 | nSBME |
| NU_ME | ASN | 259 | 6.88 | 0 | C | 1 | nSBME |
| NU_ME | ASN | 305 | -2.54 | 7.2 | C | 2 | nSBME |
| NU_ME | ASN | 477 | 2.75 | 24.5 | C | 1 | nSBME |
| NU_ME | ASN | 525 | -1.42 | 27 | H | 1 | nSBME |
| NU_ME | ASN | 555 | 1.98 | 1.4 | H | 1 | nSBME |
| NU_ME | ASN | 619 | -1.76 | 28.6 | H | 1 | nSBME |
| NU_ME | ASP | 135 | 2.83 | 59.7 | C | 1 | nSBME |
| NU_ME | ASP | 181 | -2.79 | 54.9 | C | 1 | nSBME |
| NU_ME | ASP | 218 | 1.61 | 83 | H | 1 | nSBME |
| NU_ME | ASP | 256 | 9.32 | 34.2 | C | 2 | nSBME |
| NU_ME | ASP | 33 | 1.05 | 53.6 | H | 1 | nSBME |
| NU_ME | ASP | 627 | 0.91 | 79.2 | C | 1 | nSBME |
| NU_ME | GLN | 17 | -5.72 | 38.2 | S | 1 | nSBME |
| NU_ME | GLN | 219 | -3.59 | 28.3 | H | 1 | nSBME |
| NU_ME | GLU | 169 | 0.79 | 72.1 | S | 1 | nSBME |
| NU_ME | GLU | 221 | 1.58 | 69 | H | 1 | nSBME |
| NU_ME | GLU | 239 | -1.47 | 45.4 | S | 3 | nSBME |
| NU_ME | GLU | 332 | 0.04 | 25.3 | C | 2 | nSBME |
| NU_ME | GLU | 339 | -2.92 | 26.4 | S | 3 | nSBME |
| NU_ME | GLU | 55 | 1.05 | 60.3 | H | 1 | nSBME |
| NU_ME | GLU | 609 | 0.77 | 50.5 | H | 1 | nSBME |
| NU_ME | GLY | 216 | -0.85 | 0 | C | 1 | nSBME |
| NU_ME | GLY | 244 | 1.78 | 0 | C | 1 | nSBME |
| NU_ME | GLY | 288 | 0.87 | 0 | C | 1 | nSBME |
| NU_ME | GLY | 472 | -0.84 | 0 | C | 1 | nSBME |
| NU_ME | GLY | 474 | -2.27 | 0 | C | 2 | nSBME |
| NU_ME | GLY | 475 | -4.21 | 0 | C | 1 | nSBME |
| NU_ME | GLY | 506 | -2.44 | 0 | C | 2 | nSBME |
| NU_ME | GLY | 507 | -0.80 | 0 | C | 1 | nSBME |
| NU_ME | GLY | 518 | -2.17 | 0 | H | 2 | nSBME |
| NU_ME | GLY | 683 | 0.84 | 0 | C | 1 | nSBME |
| NU_ME | HIS | 79 | 0.83 | 10.1 | C | 1 | nSBME |
| NU_ME | LEU | 617 | -1.06 | 4.4 | H | 1 | nSBME |
| NU_ME | LYS | 157 | -3.64 | 41.6 | S | 1 | nSBME |
| NU_ME | LYS | 162 | -1.02 | 66.5 | S | 1 | nSBME |
| NU_ME | LYS | 209 | -0.80 | 21.4 | S | 1 | nSBME |
| NU_ME | LYS | 233 | -0.80 | 54.9 | C | 1 | nSBME |
| NU_ME | LYS | 449 | -1.59 | 52.6 | S | 1 | nSBME |
| NU_ME | LYS | 516 | -9.73 | 61.9 | H | 2 | nSBME |
| NU_ME | LYS | 665 | -0.79 | 25 | C | 1 | nSBME |
| NU_ME | LYS | 75 | 1.23 | 19.1 | S | 1 | nSBME |
| NU_ME | LYS | 84 | -4.72 | 68.7 | C | 1 | nSBME |
| NU_ME | PHE | 173 | -1.39 | 18 | C | 1 | nSBME |
| NU_ME | PHE | 476 | 4.33 | 11.2 | C | 1 | nSBME |
| NU_ME | PRO | 165 | -2.02 | 86.5 | S | 1 | nSBME |
| NU_ME | PRO | 257 | -3.39 | 8.2 | C | 1 | nSBME |
| NU_ME | PRO | 27 | -2.24 | 14.8 | C | 1 | nSBME |
| NU_ME | PRO | 451 | -1.90 | 0.2 | S | 1 | nSBME |
| NU_ME | PRO | 568 | -1.54 | 11.3 | H | 1 | nSBME |
| NU_ME | PRO | 616 | -2.60 | 0 | H | 1 | nSBME |
| NU_ME | PRO | 631 | -0.90 | 0 | C | 1 | nSBME |
| NU_ME | SER | 144 | -6.03 | 2.7 | S | 1 | nSBME |
| NU_ME | SER | 308 | 1.00 | 0.8 | C | 1 | nSBME |
| NU_ME | SER | 444 | -20.84 | 0.2 | C | 1 | nSBME |
| NU_ME | SER | 615 | 4.02 | 0 | H | 1 | nSBME |
| NU_ME | SER | 694 | -1.70 | 0 | H | 1 | nSBME |
| NU_ME | THR | 200 | -5.74 | 20 | C | 1 | nSBME |
| NU_ME | THR | 217 | 1.61 | 34 | C | 1 | nSBME |
| NU_ME | THR | 304 | -1.95 | 0.4 | S | 2 | nSBME |
| NU_ME | THR | 448 | -11.73 | 31.7 | C | 1 | nSBME |
| NU_ME | THR | 597 | 2.64 | 18.2 | H | 1 | nSBME |
| NU_ME | TRP | 326 | 0.81 | 6.2 | H | 1 | nSBME |
| NU_ME | TYR | 187 | -14.25 | 1.2 | S | 1 | nSBME |
| NU_ME | TYR | 290 | 2.11 | 2 | S | 1 | nSBME |
| NU_ME | TYR | 311 | 1.00 | 6.3 | C | 1 | nSBME |
| NU_ME | TYR | 473 | -1.09 | 15.6 | C | 1 | nSBME |
| NU_ME | TYR | 589 | -1.10 | 10.1 | H | 1 | nSBME |
| NU_ME | TYR | 599 | -0.91 | 0.5 | H | 1 | nSBME |
| NU_ME | TYR | 614 | 0.87 | 2.9 | H | 1 | nSBME |
